# Oxidative Stress and Inflammation in Acute and Chronic Lung Injuries

**DOI:** 10.3390/antiox12030548

**Published:** 2023-02-21

**Authors:** Frank Silva Bezerra, Manuella Lanzetti, Renata Tiscoski Nesi, Akinori Cardozo Nagato, Cyntia Pecli e Silva, Emanuel Kennedy-Feitosa, Adriana Correa Melo, Isabella Cattani-Cavalieri, Luís Cristóvão Porto, Samuel Santos Valenca

**Affiliations:** 1 Department of Biological Sciences, Federal University of Ouro Preto, Ouro Preto 35400-000, Brazil; 2 Institute of Biomedical Sciences, Federal University of Rio de Janeiro, Rio de Janeiro 21941-902, Brazil; 3 Advanced Cardiopulmonary Education and Rehabilitation Center, Criciúma 88806-000, Brazil; 4 Department of Physiology, Federal University of Juiz de Fora, Juiz de Fora 36036-900, Brazil; 5 Institute of Biophysics Carlos Chagas Filho, Federal University of Rio de Janeiro, Rio de Janeiro 21941-617, Brazil; 6 Department of Health Sciences, Federal University of the Semi-Arid Region, Mossoró 59625-900, Brazil; 7 Department of Physical Therapy, Badim Hospital, Rio de Janeiro 20550-013, Brazil; 8 School of Pharmacy, Chapman University, Orange, CA 92866, USA; 9 Institute of Biology Roberto Alcântara Gomes, Rio de Janeiro State University, Rio de Janeiro 20950-003, Brazil

**Keywords:** asthma, chronic obstructive pulmonary disease, emphysema, hyperoxia, pulmonary fibrosis, sepsis, ventilator-induced lung injury

## Abstract

Acute and chronic lung injuries are among the leading causes of mortality worldwide. Lung injury can affect several components of the respiratory system, including the airways, parenchyma, and pulmonary vasculature. Although acute and chronic lung injuries represent an enormous economic and clinical burden, currently available therapies primarily focus on alleviating disease symptoms rather than reversing and/or preventing lung pathology. Moreover, some supportive interventions, such as oxygen and mechanical ventilation, can lead to (further) deterioration of lung function and even the development of permanent injuries. Lastly, sepsis, which can originate extrapulmonary or in the respiratory system itself, contributes to many cases of lung-associated deaths. Considering these challenges, we aim to summarize molecular and cellular mechanisms, with a particular focus on airway inflammation and oxidative stress that lead to the characteristic pathophysiology of acute and chronic lung injuries. In addition, we will highlight the limitations of current therapeutic strategies and explore new antioxidant-based drug options that could potentially be effective in managing acute and chronic lung injuries.

## 1. Introduction

Population growth associated with a 21st-century lifestyle has opened a window to a wide range of diseases that currently threaten human health [1]. The global burden of acute and chronic lung injuries in adults and children is constantly increasing [2]. The cause of mortality and morbidity from many respiratory diseases is unclear. However, recent statistics published by the World Health Organization (WHO) estimate that 1 billion people of the world’s population will suffer from acute and/or chronic lung diseases in the next 10 years due to air and/or environmental pollution exposure, infection (viruses, bacteria, and other pathogenic microorganisms), or specific genetic backgrounds (e.g., as for cystic fibrosis and α1-antitrypsin deficiency) [3].

The lungs are one of the few internal organs that are exposed to a wide range of environmental pollutants that include organic/inorganic/biological agents from diverse natural and anthropogenic sources and are under constant threat of developing simple to complex disorders, which can compromise the quality of life and, ultimately, even lead to death [4]. Based on the pathophysiology and mode of transmission, acute and chronic lung injuries can be broadly categorized into infectious (such as pneumonia) and non-infectious diseases (e.g., pulmonary fibrosis) [5]. These respiratory pathologies have a varied set of causes and are diagnosed very differently. However, most non-infectious diseases are typically treated similarly. These treatments usually include bronchodilators, corticosteroids, and antibiotics [6] that only target the main symptoms of the disease, without offering any long-term relief, neither in terms of prevention nor cure. Continuous advancements in understanding the underlying mechanisms (involving molecular and immunological pathways) that drive lung injuries can lead to the identification of promising therapeutic targets. In fact, our knowledge of the molecular pathology of these diseases has considerably improved over the last two decades after the genome project conclusion [7]. Regions with a substantial burden of lung diseases lack proper prevention/management strategies, which is of particular concern in low- and middle-income countries such as India, China, and those in Latin America, Africa, and the Asia-Pacific [8]. This review will discuss the main acute and chronic lung injuries that currently threaten the health of millions of people worldwide.

## 2. Oxidative Stress and Inflammation in Asthma

Asthma is an airway disease characterized by chronic inflammation and airway hyperreactivity (AHR). Its pathophysiology involves excessive airway bronchoconstriction, accumulation of immune cells (such as lymphocytes and eosinophils), mucus hypersecretion, pro-inflammatory mediators (including cytokines, histamine, and prostanoids), and reactive oxygen species [9,10]. Clinically, asthma presents as recurrent episodes of cough, dyspnea, presence of wheezing on pulmonary auscultation, and retrosternal oppression. These episodes are caused by an allergic response resulting in clinical exacerbations in the airways, with inflammatory characteristics that reach the bronchial region and contribute to airflow obstruction [11,12,13]. AHR is defined as an exacerbated response of the airways to an allergic stimulus that activates several inflammatory, oxidative, and immunological mediators, leading to bronchoconstriction and mucus hypersecretion. This condition, therefore, acutely results in a variable airflow limitation and chronically compromises the pulmonary functional capacity [14,15].

Despite proper diagnosis, classification of severity, and management of asthma in clinical situations, there is still no consensus on the mechanisms underlying AHR in asthma [16]. AHR in asthma can be divided into two categories: persistent and variable. In both situations, patients exhibit airway inflammation as well as structural and functional changes culminating in airway remodeling [17]. In asthma, the extracellular matrix, airway epithelium, and airway smooth muscle have been recognized as fundamental components of the remodeling process [18]. Structural changes in asthma include epithelial damage, mucosal gland hyperplasia, deposition of collagen and proteoglycans in the basement membrane, angiogenesis, and increased air-way smooth muscle mass [19].

Airway smooth muscle cells are the major effectors that control tone and caliber of the small airways. Histopathological studies indicate an increase in airway smooth mass in the bronchial tree due to hyperplasia and hypertrophy. Thus, structural and functional changes of these cells form a basis of the AHR in asthma [20,21].

Inflammation in asthma may be responsible for the imbalance between oxidative stress and antioxidant defense. The inflammatory response is predominantly mediated by eosinophils, neutrophils, and macrophages, as well as mediator release by airway resident cells such as mesenchymal cells (i.e., fibroblasts and airway smooth muscle cells). In particular, eosinophils have the ability to generate reactive oxygen (ROS) and reactive nitrogen species (RNS) from their granules [22]. In addition, eosinophils harbor eosinophil peroxidase (EPO), which has an affinity for bromine, forming hypobromous acid, widely known as the major cause of tissue damage exerted by eosinophils [23]. In the presence of hydrogen peroxide (H_2_O_2_), neutrophils produce the strong oxidant hypochlorous acid (HOCl) through the action of myeloperoxidase (MPO) with chlorine. Although HOCl production by MPO is considered important in the defense against pathogens, overproduction during chronic inflammation promotes host tissue damage, which is strongly linked to the progression of many inflammatory diseases [24,25].

Enzymatic and non-enzymatic systems are affected by asthma. Disturbances in total, oxidized, and reduced glutathione (GSH) have been reported in the airways of asthma patients, suggesting that GSH synthesis and/or transport is altered in response to oxidative stress [26]. Furthermore, the enzymatic activities of superoxide dismutase (SOD), glutathione peroxidase (GPx), and catalase (CAT) are affected [27].

Inflammatory cells are paramount in the pathophysiology of asthma, especially with regards to the extent of oxidative stress. For example, macrophages play a central role by generating considerable amounts of ROS and RNS [28,29], which is typically associated with an increase in pro-inflammatory cytokines such as tumor necrosis factor-alpha (TNF-α) and interleukin 1 beta (IL-1β) [30,31]. In macrophages, the respiratory burst increases the activity of inducible nitric oxide synthase (iNOS) and nicotinamide adenine dinucleotide phosphate (NADPH) oxidase, thereby increasing nitric oxide (NO^•^), peroxynitrite (ONOO^•^), superoxide (O_2_^•−^), and H_2_O_2_ [32,33]. Eosinophils release eosinophilic extracellular traps (EETs) that are rich in EPO and release H_2_O_2_ [34]; both EET formation and EPO activity rely on the generation of ROS and RNS as well as the state of oxidative stress [35]. Epithelial cells are also major producers of ROS and RNS and contribute to oxidative stress in asthma through the release of NO and chemokines such as IL-8 that recruit immune cells to the airways [36]. Importantly, neutrophil infiltration in the lungs of asthmatics raises the levels of chemokines such as motif chemokine ligand 1 (CXCL1) and CXCL8 [37]. This contributes to the maintenance of the oxidative state by MPO [38], which can produce H_2_O_2_ and extracellular neutrophil traps (NETs). Although necessary for pathogen elimination, NETs are major contributors to inflammation and oxidative stress [39,40].

### 2.1. The Role of Oxidative Stress in Asthma Pathophysiology

A complex interaction between cells and numerous pro-inflammatory mediators is evident in the pathophysiology of asthma. Among these mediators, endogenous ROS and RNS are strongly suggested to be responsible for the inflammation of the airways, and the imbalance between reducing and oxidant systems, rendering a dominant oxidative state, is a determining factor of disease severity [41,42,43,44]. Recently, increased levels of direct and indirect markers of oxidative stress have been demonstrated in asthma; these include H_2_O_2_, malondialdehyde (MDA), thiobarbituric acid reactive substances, and isoprostanes as assessed in urine, plasma, sputum, bronchoalveolar lavage (BAL), and lung tissues [45].

Considering air pollutants are rich sources of ROS, genes involved in the oxidative stress mechanism can have potent effects on the respiratory system. Because environmental factors are involved in asthma, epigenetics have been used to explain the etiology, phenotypes, and heterogeneity of this disease [46]. There is evidence to suggest that prenatal exposure to tobacco smoke, a rich source of ROS, is associated with impaired respiratory function, asthma, and/or respiratory infections in infants, young children, and adolescents [47]. Regarding asthma specifically, the interactions between genetic polymorphisms and environmental exposures suggest an epigenetic mechanism for this disease [48], beyond genomic imprinting, histone modification, altered DNA methylation, and regulation by microRNAs [49]. The increase in inflammatory cells and resulting increased local ROS production in asthmatic airways [41] involves the activation of epithelial cells and can be triggered by exposure to exogenous ROS from sources such as cigarette smoke (CS), air pollution, mites, and dust, which may lead to asthma attacks [50,51].

Allergen exposure in allergic asthma promotes the activation of leukocytes, especially eosinophils and neutrophils, and NADPH oxidase; this induces the production of O_2_^•−^ (among other ROS) and its subsequent dismutation to H_2_O_2_ [41]. ROS can directly influence airway cells and induce/promote many of the pathophysiological features associated with asthma, including lipid peroxidation, arachidonic acid release from cell membranes, alteration of protein structures, hyperresponsiveness of airway smooth muscle, increased mediator secretions in the airways, increased vascular permeability, synthesis and release of chemoattractants, decreased cholinesterase activities, and impaired adrenergic receptor responses [52,53].

**Figure 1:**
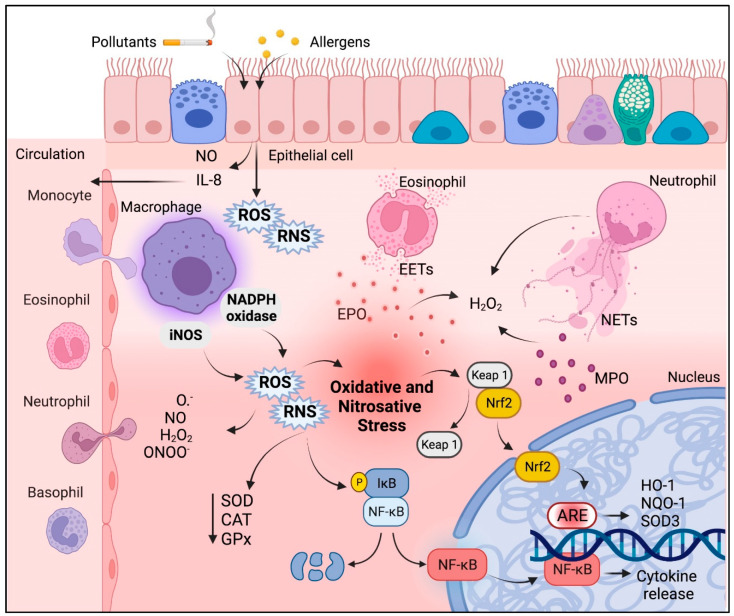
Schematic representation of oxidative and nitrosative stress in the pathophysiology of asthma. Recruitment (e.g., via IL-8) and activation of inflammatory cells such as neutrophils, macrophages, and eosinophils result in the release of ROS and RNS as well as NETs + MPO (from neutrophils) and EETs + EPO (from eosinophils), which cumulatively increase H2O2 levels. Macrophages induce the activity of iNOS and NADPH oxidase, resulting in increased release of ROS and RNS, thereby promoting oxidative/nitrosative stress and reducing the activity of antioxidant enzymes. In an attempt to counteract oxidative/nitrosative stress, cells respond to these conditions by activating the transcription factors Nrf2 and NF-κB, which will translocate to the nucleus and transcribe antioxidant enzymes and cytokines, respectively. Heme oxygenase (HO-1); hydrogen peroxide (H_2_O_2_); inducible nitric oxide synthase (iNOS); inhibitor of kB (ikB); kelch-like ECH-associated protein 1 (Keap 1); eosinophil peroxidase (EPO); myeloperoxidase (MPO); nicotinamide adenine dinucleotide phosphate (NADPH); nuclear factor erythroid 2-related factor 2 (Nrf2); nuclear factor-κB (NF-κB); nitric oxide (NO); peroxynitrite (ONOO^−^); quinone oxidoreductase (NQO1); reactive nitrogen species (RNS); reactive oxygen species (ROS); superoxide (O^•−^); superoxide dismutase (SOD), and superoxide dismutase 3 (SOD3).

Redox imbalance in asthma is also indicated by changes in nuclear factor erythroid 2-related factor 2 (Nrf2) function. In general, the initial antioxidant response protects against cellular inflammation and cytotoxic damage, whereas phase II antioxidant enzymes depend on Nrf2 regulation and can protect the cell and extracellular milieu from oxidative stress by upregulation of heme oxygenase 1 (HO-1), SOD3, NAD(P)H quinone dehydrogenase 1 (NQO1), glutathione S transferase (GST), and glutamate cysteine ligase [54,55]. These enzymes protect already inflamed airways against the pro-oxidative effects of electrophilic chemicals from pollutants such as particulate matter, considered a major contributor to asthma exacerbations [56]. Therefore, in patients with asthma, reduced levels and activation of phase II enzymes increase the susceptibility to these environmental irritants, leading to an exaggerated airway inflammatory response [57,58,59]. Thus, these enzymes play a crucial role in maintaining cellular redox homeostasis and preventing airway inflammation as well as asthma exacerbations [60,61,62]. Figure 1 summarizes the involvement and interplay of oxidative stress and inflammation in asthma.

### 2.2. Most Common Experimental Models of Asthma for the Study of Oxidative Stress

Several experimental murine models of asthma to study the inflammatory and morphological manifestations of the disease have been developed using various allergens, including ovalbumin (OVA) and pollen-derived environmental allergens [63,64]. In addition to the impact of different allergic stimuli, the type of induction also influences the experimental outcomes of these models.

Acute models of the allergic response have been widely utilized to clarify simpler mechanisms of short-term inflammatory responses, immunoglobulin E (IgE) levels, and morphological changes such as epithelial hyperplasia and hypertrophy. The majority of inflammatory cells typically observed in these acute models consist of eosinophils, and many of the changes seem to be reversed within a few days after cessation of allergen administration, not mimicking the chronic character of the disease [65]. Additional protocols make use of an adjuvant (e.g., aluminum hydroxide [HA]), which promotes a robust type 2 immune response (Th2) triggered by the immune system to more accurately reflect the clinical situation in patients [66]. Classic models of OVA + HA are still widely used, and several protocols that include allergic sensitization and allergen challenge with multiple variants of this model have been well documented [67,68].

Following sensitization with OVA + HA and subsequent OVA challenge, an increase in IgE, influx of immune and inflammatory cells, and bronchoconstriction are typically observed 24–72 h post-challenge. The number of days of sensitization and challenge varies among studies and, in addition to the route of OVA/HA administration (e.g., subcutaneous, intraperitoneal, intranasal, or aerosol inhalation), can greatly affect the allergic response and other experimental outcomes [68,69,70]. A main advantage of the OVA model is its compatibility with different strains of mice, although C57BL/6 and BALB/c are most commonly used. These models are suitable for the characterization of allergen-induced changes in lung histoarchitecture as airway obstruction, bronchial constriction, hypersecretion and AHR properties such as lung resistance and compliance over time [71].

### 2.3. Therapeutic Strategies in the Treatment of Asthma

Several therapeutic strategies are used for the treatment of asthma worldwide. Currently, the cornerstone pharmacological treatment for asthma consists of a combination of corticosteroids (anti-inflammatory agents) and β2-adrenergic agonists (bronchodilators). Whereas asthma has an important component of oxidant/antioxidant imbalance in its pathophysiology, antioxidants studied so far are not (yet) included in therapeutic strategies as clinical data are currently insufficiently available [72]. Although the drugs described above have potent effects when used alone or in combination, they often exhibit side effects or reduced effectivity over time (e.g., desensitization) that limit their long-term use [73]. Antioxidant therapy, whether through direct or indirect mechanisms, has emerged as one of the main and most promising approaches to address oxidative stress-induced cellular damage [74]. Indeed, several (preclinical) studies have already demonstrated the therapeutic potential of antioxidants, particularly with regards to asthma. It should be noted, however, that modulation of oxidative stress alone may not be as effective as its combination with other therapeutic interventions. In the following section, we will discuss various antioxidants considered promising for asthma treatment.

### 2.4. Natural Products and Bioactives

Natural extracts derived from plants contain several compounds with antioxidant properties, including benzoic acid, cinnamic acid, coumarin, gall tannic acid, and flavonoids. Flavonoids have diverse biological effects that include antioxidant, anti-inflammatory, anticancer, anti-obesity, antidiabetic, immunomodulatory, and anti-allergic properties [75,76]. As powerful antioxidants, flavonoids are able to protect cellular components against ROS by stabilizing ROS by reacting with radical compounds, scavenging NO, and inhibiting xanthine oxidase activity [9]. Accordingly, flavonoids are well known to have beneficial biological activities in respiratory tract diseases. For example, the flavonoid sakuranetin has been shown to be effective in attenuating AHR, decreasing 8-isoprostane and Th2 pro-inflammatory cytokines, as well as IgE levels, and reducing vascular peribronchial and lung parenchyma remodeling (by inhibiting NF-κB activation) in murine models of asthma [77,78].

Quercetin (3,5,7,3,4-pentahydroxyflavone) is a flavonoid commonly present in various consumables, such as apples, onions, potatoes, broccoli, and teas [79], as well as certain plant species [80]. It has been recognized for its antioxidant and cytoprotective capacity [81]. Some demonstrated pharmacological properties of quercetin include anticancer and antiviral activities, reduction of cell proliferation, prevention of platelet aggregation, stabilization of immune cells, relaxation of vascular smooth muscle, and protection against low-density lipoprotein oxidation [82,83]. Recently, the effects of quercetin on the airways have been demonstrated as well [84].

Resveratrol is a polyphenolic compound mainly found in peanuts, grapes (red wine), mulberries, and several plants, and has recently been proposed as a promising bioactive due to its low toxicity and broad biological activity. Several antioxidant mechanisms of resveratrol have been described, including its abilities to inhibit the production of ROS by inflammatory cells [85], scavenge free radicals, and stimulate the synthesis of endogenous antioxidants through the stimulation of ROS-related Nrf2 [86]. Beneficial effects of resveratrol have been observed in pulmonary fibrosis, chronic obstructive pulmonary disease (COPD), and pulmonary hypertension [87,88,89]. In addition, resveratrol was able to reduce airway inflammation and hyperresponsiveness in an acute asthma model [90] and induced GSH synthesis, attenuated oxidative stress, and depleted GSH in asthmatic lung epithelial cells [91]. The p47phox subunit of NADPH oxidase is an essential component for the generation of superoxide. Resveratrol treatment of obese asthmatic mice reduced OVA-induced lung inflammation concomitant with a reduction of p47phox expression and ROS production as well as an elevation of SOD levels in lung tissue [92].

Terpenes and their synthetic derivatives have been reported to exhibit diverse pharmacological activities, including antifungal, antiviral, antibacterial, antiarrhythmic, antispasmodic, antihistaminic, anti-inflammatory, and antioxidant effects. The latter property has been attributed to the ability of these compounds to modulate the endogenous antioxidant system and directly eliminate ROS [74].

### 2.5. Diet and Supplements

Foods and nutrients play an important role in the protection of the airways and lungs against oxidative damage. This is evident, for example, from the observation that reduced consumption of fruits and vegetables results in decreased levels of defense antioxidants, thereby increasing the susceptibility to inhaled irritants [93,94,95]. Furthermore, low intake of fruits, vegetables, juices, and vitamins (A, C, and E) was linked to deficits in lung function in children [96], and an association between low serum vitamin A levels and airway obstruction has been shown in healthy adults [97]. Because antioxidant enzymes are fundamental in the defense of the airways against oxidative stress, some proposed therapeutic strategies involve the intake of micronutrients such as vitamins (A, C, and E), polyphenols, and carotenoids to help protect (asthmatic) individuals from oxidative stress and airway inflammation [98,99,100,101,102,103,104,105].

Vitamin A and carotenoids have been reported to be beneficial in several human diseases, including diarrhea, acute respiratory infection, ischemic heart disease, immune disorders, and asthma [106,107,108]. These effects might be associated with the known preventative actions of carotenoids (e.g., β-carotene) on lipid peroxidation and DNA damage. Beta-carotene is abundant in plants and fruits and is a precursor to vitamin A. It can act on membranes, eliminate the superoxide anion, and react directly with peroxyl free radicals, serving as a soluble antioxidant [109].

Vitamin E collectively refers to a group of vitamins found in a variety of foods with antioxidant properties, of which α-tocopherol has been studied the most. It can protect the cell membrane by interfering with the lipid peroxidation chain reaction. Although supplementation with α-tocopherol had no effects on FEV1 in patients with mild to moderate asthma, studies in Finland and Italy reported some beneficial effects on lung function as α-tocopherol reduced wheezing in asthmatic adults [110].

Vitamin C is the most abundant antioxidant in the pulmonary extracellular fluid. Its antioxidant activity is exerted through distinct mechanisms such as scavenging oxygen free radicals and suppressing the secretion of superoxide anions by macrophages [111]. Furthermore, vitamin C reacts with and deactivates free radicals before they can damage proteins and lipids by donating hydrogen, thereby preventing/reversing oxidation [112].

Vitamin D is derived from food intake or can be synthesized following exposure to sunlight. Patients with severe asthma who are vitamin D-deficient have been shown to have lower FEV1 values compared to those with sufficient vitamin D. In addition, the absence of vitamin D3 results in increased production of ROS and consequent DNA damage, which could be significantly decreased by vitamin D3 supplementation [113]. In a murine model of OVA-induced asthma, treatment with vitamin D3 showed protective effects by reducing α-smooth muscle actin expression, airway inflammation, collagen deposition, goblet cell hyperplasia, as well as TGFβ/Smad signaling, and increased activation of the Nrf2/HO-1 pathway [114].

### 2.6. Enzyme Mimetics and Bioactives

Generally, enzyme mimetics are considered small compounds that have the catalytic capacity to mimic the natural activity of larger enzymes such as SOD, CAT, and GPx [45]. SOD activity is impaired in the airways of patients with asthma in the presence of oxidants and is even further reduced following exacerbations of asthmatic crises due to the high production of oxygen radicals by inflammatory cells [59]. Therefore, strategies that aim to increase SOD levels in the airways could be promising and effective for asthma treatment [115,116]. Ideally, SOD mimetics should exhibit properties such as scavenging O_2_^•−^, H_2_O_2_, ONOO^•^, and lipid peroxides [115]. SOD mimetics developed based on organomanganese complexes such as AEOL10150 and AEOL10113 have shown beneficial effects on airway inflammation. For example, AEOL10113 inhibited bronchial inflammation and hyperreactivity in an OVA mouse model of asthma [115]. A GPx mimetic has shown promise as an antioxidant by reducing levels of various ROS; these included singlet oxygen (a more reactive form of oxygen), HOCl, and ONOO^•^ [117].

NADPH oxidase is responsible for the production of O_2_^•−^ and, indirectly, the generation of other ROS and RNS [118]. Excessive production of ROS by NADPH oxidase is thought to play a crucial role in tissue damage associated with several inflammatory respiratory diseases, including cystic fibrosis, COPD, and asthma [119], and has therefore been considered a possible target in antioxidant therapy. Apocynin is a natural organic compound and antioxidant that blocks NADPH oxidase activation, likely by reacting with its thiol compounds. Inhaled administration of apocynin did not change respiratory parameters such as FEV1 and peak expiratory flow in asthmatic patients [120]. However, it was able to reduce H_2_O_2_, nitrite (NO_2_^−^), and nitrate (NO_3_^−^) levels in exhaled air condensate, indicating its anti-inflammatory potential [121].

The relationship between pathophysiology and several antioxidant treatments for asthma is described here. A main advantage of using natural compounds for the treatment of asthma is the low side effects; however, more translational studies in this field are necessary to validate the use of natural antioxidants alone or in combination with other therapeutics as an option for patients.

## 3. Oxidative Stress and Inflammation in COPD and Emphysema

The lungs are constantly exposed to potentially harmful particles that can disrupt the homeostasis of the cells that cover this organ, which may culminate in irreversible morphological and functional changes [122]. Several inflammatory and oxidative events have been reported as key factors for COPD, whether causing bronchoconstriction, pulmonary emphysema, or a range of pathophysiological alterations that are indicative of the heterogeneity among COPD patients [123]. Furthermore, disease progression is typically characterized by increased small-airway fibrosis [124].

Cigarette smoke (CS) is the main risk factor for COPD and is composed of numerous toxic chemicals, including ROS. Excessive ROS levels induce oxidative stress, leading to deleterious changes in cellular molecules such as proteins, lipids, and DNA [125,126]. In COPD, inflammation is persistent even after smoking cessation, resulting in continuous inflammatory tissue damage [123]. Various agents contribute to this tissue inflammation-related lung injury as cells and mediators of innate and adaptive immunity. The exaggerated production of ROS and RNS initiates two critical mechanisms: the imbalances between proteases/antiproteases and oxidants/antioxidants [127]. CS and other irritating inhalants trigger both innate and adaptive immune responses, leading to the expression and activation of inflammatory mediators that damage local components, such as lung epithelial cells, fibroblasts, and ECM elements (e.g., elastic and collagen fibers). Ultimately, these alterations lead to a loss in lung compliance, which characterizes the COPD phenotype [128].

### 3.1. Proteolytic Imbalance

The connective tissue in the lung forms the pulmonary scaffold, organized by the ECM. The basement membrane, the airway lamina propria, and the alveolar interstitium (a thin compartment between the alveoli and the blood vessels) constitute the main structures of the lung ECM. The primary components of the basement membrane are collagen IV and laminin, while the lamina propria and interstitium predominantly consist of fibrillar collagens, elastic fibers, fibronectin, glycosaminoglycans, and proteoglycans [129,130]. ECM composition is tightly regulated by endogenous proteins such as MMPs and their inhibitors (tissue inhibitors of metalloproteinases [TIMPs]); the balance between MMPs and TIMPs is essential for ECM homeostasis [131].

MMPs are proteolytic enzymes, which constitute a large family of zinc-dependent proteinases and are generally synthesized and secreted by macrophages as well as structural cells of the lungs in response to activation by various mediators. However, MMPs can also be produced/stored in neutrophils and released upon degranulation [132]. MMPs can be categorized into distinct subclasses such as gelatinases (MMP2 and 9), collagenases (MMP1, 8, and 13), type-membrane MMPs (MMP14 to MMP25), and macrophage MMP (MMP12) [133]. CS exposure leads to the recruitment of neutrophils and macrophages, which can produce large amounts of proteases, thereby disrupting the balance between MMPs and TIMPs. This results in proteolysis, particularly affecting elastin, and tissue destruction [134].

Several studies have shown a reduction in elastic fibers as well as ruptured fibers and impaired elastogenesis in COPD. Patients with severe COPD have been reported to exhibit upregulation of several genes related to elastogenesis, including elastin and fibulin-5, in lung tissue [135,136]. Fibulin-5 knockout mice appear to modulate the formation of elastic fibers, leading to an elastinopathy resembling a severe form of emphysema [137]. The role of proteolysis has been established and recognized since emphysema was first described as a disease associated with an antiprotease deficiency [138]. As indicated, CS or irritants derived from polluted air attract inflammatory cells and create an overwhelming proteolytic environment, primarily comprising neutrophil-derived proteases and elastases, macrophage-derived MMP12 [139], cathepsins L and S [140], and collagenases MMP2 and MMP9 [141]. The antiproteolytic shield is mainly composed of α1-antitrypsin (AAT), a neutrophil elastase inhibitor, and TIMPs [142]. In addition to ECM degradation due to proteolytic imbalance, resultant ECM fragments can act as chemokines and promote local inflammation [143]. Specifically, laminin and fibronectin fragments are chemotactic for human neutrophils and monocytes [144]. Thus, ECM proteolysis generates fragments that can perpetuate inflammation regardless of current smoking status [145].

### 3.2. Redox Imbalance

**Figure 2:**
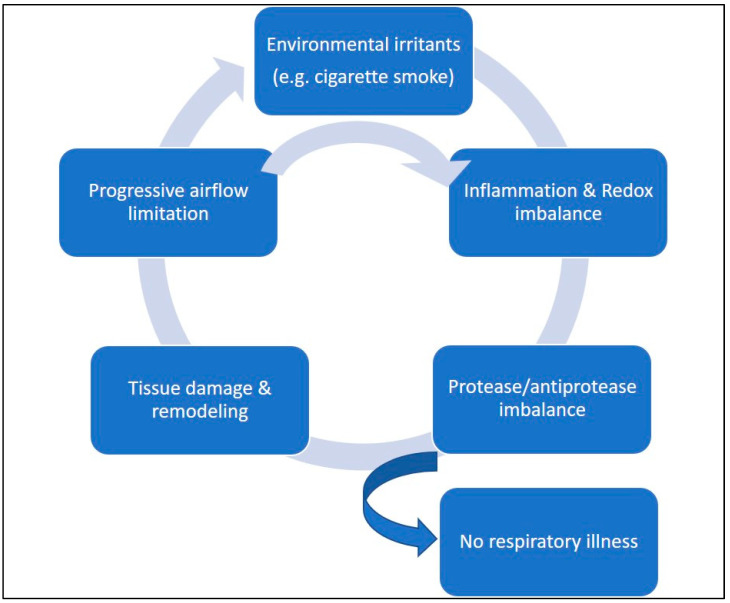
The progressive loop in COPD pathophysiology. Oxidative and toxic substances (e.g., cigarette smoke) trigger cellular and molecular changes in the lung environment—presenting as chronic inflammation—potentially leading to an insufficient repair response, disintegration of the lung parenchyma (emphysema), and a dysregulated functional response in the small airways (bronchitis). Inflammation may persist even after cigarette smoke exposure cessation. The majority of smokers escape this cycle due to largely unknown reasons, but probably resulting from a complex interaction between genes and the environment [149].

Chronic cigarette smoking exposes the respiratory tree and lungs to elevated levels of exogenous ROS, resulting in oxidative stress and the induction of cell damage processes associated with lung inflammation [125]. Inflammatory and structural cells, including neutrophils, macrophages, and epithelial cells, that are activated in the airways of COPD patients also produce ROS [146]. CS activates transcription factors such as NF-κB and activator protein 1 and leads to a post-translational modification of histone deacetylase (HDAC) in macrophages [147]. These processes activate inflammatory cells, triggering the release of endogenous ROS and pro-inflammatory cytokines, consequently leading to the recruitment of more neutrophils, macrophages, and dendritic cells and an exacerbation of the inflammatory process [148]. These steps perpetuate a cyclic and progressive condition that configures the physiopathology of COPD (Figure 2).

The imbalance generated by the load of oxidants/ROS from exogenous (e.g., CS) and endogenous (inflammatory cells) stimuli in the respiratory tract results in oxidative stress, induction of vascular endothelial cell apoptosis, and impairment of the phagocytic capacity of alveolar macrophages, ultimately leading to necrosis and emphysema [150]. Oxidative stress can also impair the function of antiproteases such as AAT and secreted leukocyte protease inhibitors, thereby accelerating the breakdown of elastin in the lung parenchyma [151].

O_2_**^•^**^−^ generated by NADPH oxidase is converted to H_2_O_2_ and subsequently water by the antioxidant enzymes SOD and CAT, respectively. In the presence of iron salts, O_2_**^•^**^−^ and H_2_O_2_ can react together to form the highly reactive hydroxyl radical (HO^•^). O_2_**^•^**^−^ can also combine with NO^•^ to form peroxynitrite, which in addition to inducing oxidative damage to proteins also generates HO^•^ by itself [152]. As indicated, CS is a source of RNS as well. NO^•^ is present in large amounts in CS and can also be generated endogenously by iNOS expressed in inflammatory cells [153]. NO^•^ reacts with O_2_**^•^**^−^ to produce nitrogen dioxide (NO_2_) and ONOO^•^, leading to nitrosative stress. Osoata et al. reported that ONOO^•^ is increased in the exhaled breath condensates of COPD patients [154].

Detrimental effects of ROS on lung tissue include direct damage to the membrane of lung parenchymal cells (lipid damage), modifications of enzymes and proteins important for cell metabolism, and mutations in DNA, resulting in apoptosis of these cells. An increase in the formation of 4-hydroxy-2-nonenal (4HNE), a product of lipid peroxidation, has been observed in both the airways and alveolar epithelial cells of COPD patients [155]. Levels of MDA, another product of lipid peroxidation, were elevated in the blood of COPD patients and increased with disease severity [156,157].

Normal production of oxidants is counteracted/balanced by several antioxidants in the human respiratory system, including CAT, SOD, and GSH (a tripeptide formed by the enzymes glutamate cysteine ligase and glutathione synthetase). Extracellular antioxidants, particularly GPx, are markedly upregulated in response to CS and oxidative stress [158]. Most antioxidants are regulated by Nrf2, which is activated by oxidative stress. When stimulated, Nrf2 translocates to the nucleus, where it binds with antioxidant response elements (ARE) and transcribes genes encoding antioxidant enzymes [159]. In the lungs of COPD patients, Nrf2 is not activated properly despite high levels of oxidative stress [160,161]. Disruption of Nrf2 favors severe emphysema and, even in the absence of exogenous irritants, oxidative stress is maintained due to the continued production of ROS from endogenous sources [162].

### 3.3. Experimental Models of Pulmonary Emphysema and COPD

The use of animal models for emphysema and COPD allows for the investigation of specific pathological features, such as bronchoconstriction and alveolar destruction, and the identification of contributing mechanisms, which ultimately facilitates the development of novel therapeutic strategies. The large majority of these models have been designed in mice and rats, although guinea pigs [163], hamsters [164], rabbits [165], and dogs [166] have been used as well.

As the primary risk factor for COPD, CS is largely employed in basic research. Other experimental stimulants include porcine pancreatic elastase (PPE), the main alternative for CS [167], papain [168], air pollution [169,170], or biomass burn [171], either alone or combined with other provocations such as lipopolysaccharide (LPS) [172]. Some protocols consider mixing CS and other stimuli to provoke an exacerbation superimposed on tissue destruction, typical of emphysema [173]. For example, in a Wistar rat model of COPD, LPS was intratracheally instilled on the first and the 14th day, and animals were challenged with CS on days 2–13 and 15–30 [88]. On the other hand, when only using cigarettes (without LPS), pathological features generally take longer to establish; to overcome this issue, the number of cigarettes can be increased with exposures taking place over 60 consecutive days (7 days a week) [174]. Alternatively, animals can be exposed for 5 days a week for 6 months [175]. Several protocols have been described and each model of induction has an ideal dose/quantity of cigarettes and CS exposure time for emphysema establishment.

The type of cigarette used to expose the animals represents another topic of discussion. Several groups use commercial cigarettes that are generally consumed by people and therefore mimic human conditions. On the other hand, some defend the use of cigarettes specifically developed for research purposes (Kentucky Research and Development Center, Lexington, KY, USA), allowing for more reliable and consistent experimental conditions. Information on particulate matter, tar, nicotine, and carbon monoxide contents is also available for most commercial cigarettes [176].

### 3.4. Antioxidant Therapeutic Strategies for COPD

Current therapies are effective (to some extent) in managing COPD symptoms but are unable to reverse or sufficiently prevent disease progression, despite the advanced understanding of the pathological mechanisms involved. Considering the pathophysiological role of oxidative stress, several groups have been exploring natural and synthetic antioxidants as therapeutic agents, alone or as adjuvants, in an attempt to inhibit emphysema establishment. Non-pharmacological treatments, including lifestyle changes in diet and physical activity, have been suggested for COPD patients. Epidemiological studies propose that diets rich in vitamin C, vitamin E, and β-carotenes (fruit, vegetables, oily fish, whole grains) are positively associated with lung function, and could protect against COPD [177,178]. This may be attributed to the ability of these micronutrients to directly combat oxidative stress and, consequently, inflammation triggered by it.

Oxidative stress is a major driver of COPD pathogenesis [179]. Thus, the high content of oxidants to which the pulmonary microenvironment is subjected and the depletion of antioxidant defense systems in response to excessive exposure contribute to (chronic) inflammation. Indeed, exogenous (e.g., from CS and air pollution) and endogenous oxidants (from respiratory burst mediated by complex NADPH oxidase in leukocytes, and from mitochondrial respiration in lung epithelial cells) are typically elevated in COPD patients [180]. Continuous oxidative stimulation leads to exhaustion of the antioxidant response and a reduction of its master regulator Nrf2 (and associated downstream signaling). In this context, investigating the ability of antioxidant supplementation as a therapeutic strategy has gained interest within the research community and could be explored to better understand the mechanisms that underpin tissue remodeling in emphysema.

**Figure 3:**
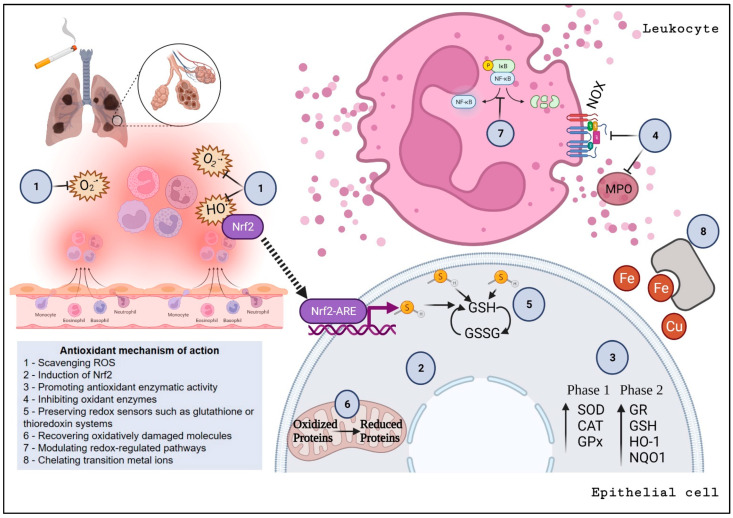
Schematic representation of various antioxidant mechanisms of action. Top left shows induction of oxidative stress and inflammation of the lung microenvironment by cigarette smoke inhalation. On the right, the roles of leukocytes and epithelial cells as well as associated signaling events are outlined. Grey circles numbered from 1 to 8 represent the pathways impacted by exogenous antioxidants and are detailed in the grey box (bottom left).

Antioxidants can boost the redox system via various specific mechanisms of action, which include (i) scavenging ROS, (ii) induction of Nrf2, (iii) promoting antioxidant enzymatic activity, (iv) inhibiting oxidant enzymes, (v) preserving redox sensors such as glutathione or thioredoxin systems, (vi) recovering oxidatively damaged molecules, (vii) modulating redox-regulated pathways, and (viii) chelating transition metal ions [181,182,183,184,185]. A schematic summarizing these antioxidant effects is presented in Figure 3.

### 3.5. Effectiveness of Synthetic Antioxidants in Emphysema/COPD

N-acetylcysteine (NAC) has been extensively studied and used as adjuvant therapy in COPD patients, reaching phase 4 in clinical trials. Beyond its mucolytic effect, NAC acts as a donor of cysteine, the limiting amino acid for GSH synthesis. In a large study with a high dose of NAC (600 mg twice daily) in over 1000 Chinese COPD patients, a reduction of around 20% in acute exacerbations was observed [186]. A more recent analysis complementing these data showed that the reduction of exacerbations by NAC was greatest in current smokers and ex-smokers and in patients receiving NAC in combination with any long-acting bronchodilator but not inhaled corticosteroids [187]. Because of such promising results, NAC has been used as a positive antioxidant control in several experimental models to identify other antioxidant strategies to treat emphysema and other COPD complications. For example, in a hybrid murine model in which emphysema was induced by porcine pancreatic elastase (PPE) and exacerbated by CS particulate matter, NAC treatment reduced CS-mediated pulmonary damage by preventing oxidative stress and reducing inflammatory responses [188]. In addition, Nrf2-GSH signaling was shown to be important for the protective effects of NAC against CS-induced injury in ATII cells. Importantly, in ATII cells deficient in Nrf2, NAC was suggested to provide partial protection through ROS-scavenging activities [189].

In a model using just PPE (a non-oxidative stimulus) to induce emphysema in mice, the role of iNOS was investigated. Following PPE-induced emphysema, mice exhibited augmented levels of nitrotyrosine, an oxidation-modified residue on proteins induced by peroxynitrite exposure. Treatment with aminoguanidine was used as a pharmacological strategy to inhibit iNOS, which resulted in a reduction in oxidative damage to proteins and protection of the lung parenchyma. The use of iNOS knockout mice corroborated these data [190]. These findings suggest that iNOS acts as an oxidant enzyme in pulmonary emphysema and may represent a target for pharmacological modulation.

Pharmacological mimetics for antioxidant enzymes also represent a potential therapeutic strategy for emphysema. Extracellular superoxide dismutase (EC-SOD or SOD3) is highly expressed in the lungs and functions as a scavenger of superoxide anion in the ECM microenvironment. SOD3 has been shown to protect against oxidative fragmentation of ECM components, such as heparin sulfate and elastin, thereby attenuating lung inflammatory responses. Positive modulation of SOD3, either in SOD3-transgenic mice or via administration of the pharmacological SOD-mimetic MnTE-2-PyP to WT or SD3-KO mice, attenuated features of emphysema and reduced oxidative fragmentation of the ECM in mouse lungs [191]. Thus, augmentation of antioxidant enzyme activity using pharmacological mimetics may have therapeutic potential in the intervention of COPD/emphysema. Additionally, another group investigated the protective role of glutathione peroxidase 1 (GPx1) against acute lung inflammation induced by CS. They used gpx-1 knockout mice and the gpx mimetic ebselen, showing gpx-1 protects against CS-induced lung inflammation and that gpx-1 mimetics may be of therapeutic interest, especially prophylactically [192].

Diallyl disulfide (Dads) exists naturally in garlic and other species of the genus Allium, but it has also been chemically commercialized and shown to protect against oxidative stress and some cancerous molecules such as nitrosamines (a carcinogen present in CS) [193]. Dads has been reported to act as an antioxidant enzyme activator (GST, SOD, GPx, and CAT), stimulator of GSH synthesis [194], and an inhibitor of cytochrome P450 family 2 subfamily E member 1, which limits the production of ROS and carcinogens [195]. Therapeutic intervention with Dads in mice blocked emphysema establishment despite continuous stimulation with CS. Moreover, the highest dose of Dads prevented tissue and oxidative damage, as indicated by similar levels of 4HNE, nitrotyrosine, and carbonyl reductase 1 in the intervention as in the sham-smoking group [196]. Protective effects of Dads have also been demonstrated in a rat model of emphysema induced by intraperitoneal injection of CS extract. Dads not only exhibited antioxidant but anti-inflammatory effects as well, increasing antioxidant enzymes, decreasing cell influx in the BAL, suppressing cytokine production, and reducing oxidative stress markers [197].

Statins constitute a class of drugs initially developed to treat metabolic disorders but have also been shown to exhibit a range of intriguing pharmacological actions, the so-called pleiotropic effects, independent of their cholesterol-lowering actions [198]. These include anti-inflammatory and antioxidant properties that have been associated with protective effects against COPD. For example, inhaled atorvastatin and simvastatin administered to emphysematous mice improved lung histoarchitecture, ameliorated lung function, reduced ROS levels, and restored SOD and CAT activity as well as the GSH/glutathione disulfide (GSSG) ratio, eliminating any increase in oxidative damage markers [199]. Following up on this study, Melo and co-workers examined the effects of atorvastatin on PPE-induced emphysema in mice. They demonstrated that atorvastatin dose-dependently promotes mouse lung repair after emphysema induced by elastase. All redox parameters evaluated, including Nrf2, SOD, CAT, GSH/GSSG, and MDA, returned to control levels in treated animals [200].

As summarized in Table 1, these reported powerful actions of synthetic antioxidants against ROS in oxidative stress-related diseases deem them promising agents in the modulation/treatment of pulmonary emphysema [201].

### 3.6. Natural Antioxidants Effective against Emphysema/COPD

Polyphenols represent an important group of molecules that have been explored in the context of pulmonary disease and other illnesses. Polyphenols are commonly present in regular diets containing fruits, vegetables, cereals, and beverages such as coffee, tea, and wine. Recently, researchers have extensively investigated dietary polyphenols and their potential chemoprotective effects on disease and the maintenance of human health.

Lanzetti and co-workers explored the benefits of mate tea (prepared from the roasted herb *Ilex paraguariensis*), a beverage very common in South America (especially in Brazil). Mate tea contains several polyphenols, with chlorogenic acid being the most abundant. The protective effects of mate tea against CS-induced emphysema were demonstrated in mice, preserving elastic fibers and lung architecture as well as the GSH/GSSG redox sensor ratio and catalase activity. Moreover, the treatment prevented pro-oxidant MPO activity induced by CS in the emphysematous group [203].

Effects of the polyphenol eucalyptol have been investigated using an experimental recovery protocol in mice with established CS-induced emphysema. Eucalyptol promoted lung repair in emphysematous mice, suggesting it can be considered a potential phytomedicine for the treatment of COPD. Furthermore, eucalyptol was effective in reducing MDA levels, a marker of lipid peroxidation, and inversely increasing SOD activity, suggesting antioxidant action through different mechanisms [204].

Propolis, a bee-metabolized resinous substance from plant sap and gums that contains several polyphenols, was studied in a similar mouse model of emphysema recovery. Results revealed that propolis promoted lung repair by shifting macrophage polarization from M1 to M2, activating a pro-resolutive response in an Nrf2-independent manner [205].

Quercetin is a member of the polyphenol family and has been demonstrated to exert antioxidant activities via different mechanisms, including scavenging free radicals [206] and upregulating ARE-Nrf2-mediated transcription activity. The latter effect occurs through inhibiting Nrf2 ubiquitination and proteasomal turnover, and by reducing Keap1 at the posttranslational level [207]. Furthermore, quercetin was effective in preventing emphysema in mice, canceling CS effects, and keeping redox parameters such as SOD and CAT activities as well as the GSH/GSSG ratio at baseline levels. Similar effects were observed on oxidative damage parameters, showing that treatment prevented the increase in MDA and carbonylated proteins [208]. Moreover, quercetin also prevented the progression of emphysema in an elastase/LPS mouse model by reducing oxidative stress (iNOS and MDA), lung inflammation (keratinocyte-derived protein chemokine (murine IL-8), MCP1, macrophage inflammatory protein 2, IL-1β), and the expression as well as activity of MMP9 and -12. These findings seemed to be modulated by Sirtuin 1 (Sirt1) as co-treatment with sirtinol, a Sirt1 inhibitor, blocked the effects of quercetin on the lung phenotype [209].

Resveratrol is a stilbenoid polyphenol that has received considerable attention in scientific and nonscientific literature. Responsible for the famous “French paradox”, the beneficial effects on cardiovascular outcomes, despite a fat-rich diet in French people, are strongly attributed to resveratrol [210]. A study using the isomer cis-resveratrol (c-RSV) to stimulate mesenchymal stem cells from mouse bone marrow, showed that c-RSV induced vascular endothelial growth facter (VEGF) expression through heat shock protein 70 (HSP70)-mediated regulation, creating a genetically modified mesenchymal stem cell (HSP-VEGFA-MSC). The protective capacity of this stem cell line to alleviate elastase-induced pulmonary emphysema was evaluated in mice. The data indicated that c-RSV elevated the nuclear content of Nrf2 and HO-1 in a dose-dependent manner. When these cells were injected into the jugular vein of mice with elastase-induced pulmonary emphysema (to induce transgene expression), clinical outcomes were ameliorated. After c-RSV treatment, significant improvements were observed in respiratory functions, and lung tissue expression levels of VEGFA, Nrf2, and SOD2 were significantly increased [211]. Wang et al. used a hybrid rat model of emphysema induced by CS exposure intercalated with LPS instillation. Animals receiving resveratrol exhibited reduced levels of MDA accompanied by increased SOD activity as well as upregulation of Sirt1 and proliferator-activated receptor-γ coactivator-1α (PGC-1α) expression. Resveratrol also reduced inflammatory parameters such as IL-6 and IL-8 serum levels. The authors proposed that the beneficial effects of resveratrol in this rat COPD model were related to the inhibition of oxidative stress and the inflammatory response, likely mediated via the activation and upregulation of the Sirt1/PGC-1α signaling pathways [88].

Curcumin belongs to the family of curcuminoids, which are phenolic pigments extracted from the plant Curcuma longa and have demonstrated anti-inflammatory, antioxidant, and anticancer activities. Due to its molecular structure, it is poorly soluble in water, which limits its clinical application. Nevertheless, curcumin has been shown to ameliorate pulmonary emphysema in rats by promoting autophagy and inhibiting endoplasmic reticulum stress through the upregulation of Sirt1 [212]. In addition, curcumin could significantly counteract the reduced cell viability and inflammation induced by CS extract (CSE) in Beas-2B cell culture by upregulating peroxisome proliferator-activated receptor gamma (PPARγ) and inhibiting NF-κB activation. In line with these in vitro findings, curcumin effectively attenuated pulmonary function decline and inflammatory responses in a rat model of CS-induced emphysema [213].

Some non-phenolic natural antioxidants deserve attention as well. Sulforaphane, an isothiocyanate derived from cruciferous vegetables such as broccoli, Brussels sprouts, and cabbages, is well known for its Nrf2-inducing activity. It has been shown in vitro that sulforaphane protected rat alveolar epithelial cells against CSE-induced oxidative injury by upregulating Nrf2 expression and reducing ROS levels [214]. In monocyte-derived macrophages (MDMs) from COPD patients, pretreatment with sulforaphane significantly suppressed the expression of toll-like receptors 2 (TLR2), TLR4, and myeloid differentiation primary response 88 (Myd88) induced by specific agonists, culminating in the reduction of IL-6 and TNF-α levels [215]. Although TLRs are typically involved in pathways of the inflammatory response, their activation also contributes to ROS release and redox homeostasis [216].

As indicated earlier, vitamins represent a group of classical antioxidants. Vitamin C was shown to not only prevent CS-induced emphysema in senescence marker protein-30 knockout mice, which cannot synthesize vitamin C, but also provide partial pulmonary restoration after emphysema establishment. Mechanistically, vitamin C treatment diminished oxidative stress, increased collagen synthesis, and improved VEGF levels in the lungs of CS-exposed mice [217].

The vitamin E isoform γ-tocotrienol with known antioxidant and anti-inflammatory effects was tested in a mouse model of CS-induced COPD. Dose-dependent benefits of γ-tocotrienol were observed in the BALF of mice exposed to CS for 2 weeks, reducing neutrophil counts and levels of cytokines, chemokines, and oxidative damage biomarkers, as well as pulmonary pro-inflammatory and pro-oxidant gene expression, and restoring lung endogenous antioxidant activities, as indicated by inhibition of nuclear translocation of signal transducer and activator of transcription 3 (STAT3) and NF-κB, and upregulation of Nrf2. In the mice exposed to CS for 2 months (rather than 2 weeks), γ-tocotrienol ameliorated bronchial epithelium thickening and destruction of alveolar sacs, thereby improving lung function. Importantly, γ-tocotrienol exhibited better anti-oxidative efficacy and protection against emphysema and lung function decline as compared to the corticosteroid prednisolone [218]. Table 2 summarizes relevant outcomes obtained by the administration of natural antioxidants in cell cultures and animal models of emphysema/COPD.

The complexity of mechanisms involved in COPD and emphysema still represents a massive hurdle in the development of treatment strategies that can effectively reverse or at least satisfactorily manage the symptoms of these diseases. Although there is accumulating evidence (primarily of a preclinical nature) for the beneficial therapeutic effects of antioxidants in COPD/emphysema, clinical validation of these agents as standalone or combination therapeutics is required.

## 4. Oxidative Stress and Inflammation in Idiopathic Pulmonary Fibrosis

**Figure 4:**
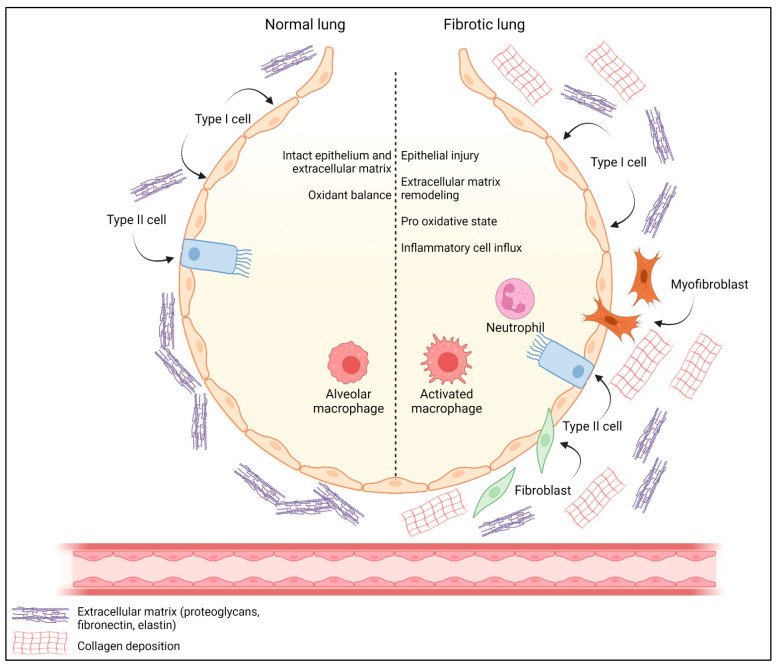
Pathogenesis of idiopathic pulmonary fibrosis (IPF). Aging, environmental, and occupational factors are associated with the development of pulmonary fibrosis. Injury to alveolar epithelial cells leads to abnormal tissue repair and extracellular matrix remodeling. Alveolar epithelial cells (type II cells) undergo epithelial-to-mesenchymal transition (EMT), promoting the accumulation, proliferation, and migration of fibroblasts and myofibroblasts. Collectively, oxidative stress and inflammation play an important role in the progression of IPF.

Idiopathic pulmonary fibrosis (IPF) is a progressive and potentially fatal disease with unknown etiology and is associated with aging as well as environmental and occupational factors (e.g., smoking) [219]. The pathogenesis of IPF is incompletely understood. However, it is well known that abnormal tissue repair and ECM remodeling play a critical role. In IPF, fibroblast migration, proliferation, and differentiation into myofibroblasts occurs. It has been proposed that epithelial to mesenchymal transition (EMT) is an important source of thses (myo)-fibroblasts [220]. Thus, alveolar epithelial cells undergo EMT, acquiring a more fibroblastic phenotype; concomitantly, proliferation and migration of fibroblasts and myofibroblasts leads to the formation of fibrotic foci (Figure 4) [221,222]. Progressive and abnormal tissue remodeling leads to modifications in pulmonary structure and function [223]. Oxidative stress and inflammation have been associated with the promotion of pulmonary fibrosis. These mechanisms, together with the protease/antiprotease imbalance, culminate in cellular apoptosis and the loss of ECM proteins.

The inflammatory process involves the infiltration of neutrophils, macrophages, and lymphocytes. Macrophages promote the development of IPF by releasing several MMPs and growth factors, including transforming growth factor beta (TGF-β), plate-let-derived growth factor (PDGF), and fibroblast growth factor (FGF) [224]. Based on phenotype, macrophages can be characterized as classically inflammatory (M1) or pro-fibrotic (M2), both of which are involved in the underlying mechanism of IPF. M2 macrophages are associated with the production of TGF-β, which is a key growth factor involved in the development of pulmonary fibrosis due to its ability to regulate ECM production and act as a modulator of the migration and differentiation of fibroblasts [225]. A recent study showed that IL-24 and IL-4 could synergistically promote the M2 phenotype [226]. PDGF is involved in lung angiogenesis and pulmonary hypertension, and elevated concentrations of PDGF have been demonstrated in alveolar macrophages from individuals with IPF [227]. The release of MMPs leads to the degradation of the ECM, rendering fragments that can promote the influx of inflammatory cells (as described on p.25). Elevated levels of several MMPs, such as MMP-3, -7, -8, and -9, have been reported in the BAL fluid of IPF patients. Moreover, the lung function in those with high levels of MMP-8 and -9 rap-idly declined over 1 year [227], suggesting that these MMPs are of particular clinical significance for the progression of IPF.

It has been suggested that the cellular redox state and the balance of oxidants/antioxidants are crucial for the progression of IPF. The lungs are more susceptible to injury by oxygen-free radicals due to their direct exposure to air [228]. Moreover, exogenous oxidants and air pollutants induce oxidant production and promote the activation of inflammatory cells, which can generate free radicals [229]. The regulation of cellular processes by ROS during normal tissue repair includes differentiation, migration, invasion, and apoptosis. However, oxidative stress promoted by excessive ROS and RNS levels leads to cellular damage and impairs normal tissue repair. The NADPH oxidase (NOX) enzymes are a primary source of ROS by catalyzing the reduction of O_2_ to form ROS. The activation of NOX isoforms has been implicated in the pathogenesis of IPF. NOX4 in particular has been associated with myofibroblast activation and phenotypic modulation of pulmonary fibroblasts [230,231,232]. These findings are supported by observations in a rodent model of bleomycin-induced pulmonary fibrosis, in which ROS production and NOX4 gene transcripts as well as protein levels were increased. Importantly, oral administration of a NOX4 inhibitor attenuated the fibrotic pathology, as indicated by reductions in fibrotic foci and levels of collagen deposition [233].

As indicated, neutrophils, macrophages, lymphocytes, fibroblasts, and myofibroblasts are important sources of ROS and RNS. Significant increases in the expression of iNOS (responsible for RNS production) and nitrotyrosine in inflammatory cells and alveolar epithelium have been observed in the lungs of patients with early to intermediate stages of IPF [234]. In serum samples, the concentrations of total hydroperoxides and 8-isoprostane (oxidative stress markers) were higher in IPF patients when compared to control subjects [235,236]. Moreover, a positive correlation between oxidative stress (total hydroperoxides) and the severity of dyspnea was demonstrated, while oxidative stress was negatively associated with the functional parameter FVC, demonstrating levels of systemic oxidative stress could serve as markers to indicate the severity of the disease [235]. Other work indicated that oxidative stress, as assessed by serum hydroperoxide, was higher in IPF patients with acute exacerbations than in those with stable disease and increased with IPF progression [237]. A study examining BAL samples demonstrated an elevation of oxidized (carbonylated) protein concentration in non-smokers with IPF compared to healthy non-smokers [238]. Taken together, there is an accumulating body of evidence to indicate a link between the modification in the cellular redox state and IPF.

Currently, there is no cure for IPF. To investigate the therapeutic potential of antioxidants, several therapeutic approaches aimed at the reduction of oxidative stress in IPF have been explored. It was recently shown that NAC treatment of rats with bleomycin- or silica-induced pulmonary fibrosis increased the activity of SOD and GSH content, significantly lowered fibrosis scores, and decreased hydroxyproline, MDA, and nitric oxide levels in lung tissue homogenates. Additionally, NAC administration reduced TGF-β, IL-β1, and TNF-α serum levels as well as the expression of PDGF and collagen deposition [239,240].

Pirfenidone and nintedanib have been approved for the treatment of IPF; however, thus far, they have not been demonstrated to satisfactorily stabilize lung function. Pirfenidone is an orally administered drug with anti-fibrotic, anti-inflammatory, and antioxidant properties that reduce the production and release of TGF-β and inflammatory cytokines while inhibiting oxidative stress [241]. Recent work demonstrated that sera from IPF patients increased intracellular ROS levels and collagen synthesis in primary human pulmonary artery smooth muscle cells (HPASMCs), which could be significantly prevented by pirfenidone treatment, suggesting antioxidant properties as the basis for the in vivo effects of this drug [242]. In primary mouse lung fibroblasts that had been induced by TGF-β1, pirfenidone improved Nrf2, HO-1, and Gpx1 mRNA and protein expression and decreased collagen I and IL-6 levels in supernatants [243]. Treatment of fibroblasts derived from IPF patients with pirfenidone was shown to inhibit the action of TGF-β1 on fibroblast proliferation and fibroblast to myofibroblast transition [244]. Nintedanib is a tyrosine kinase inhibitor that targets components of pro-fibrotic pathways, including the PDGF receptors α and β, FGF receptors 1–3, and VEGF receptors 1–3 [245]. A phase II efficacy and safety trial evaluating different doses of nintedanib (TOMORROW) [246] and placebo-controlled phase III clinical trials (INPULSIS-1 and INPULSIS-2) showed that nintedanib reduced the decline in FVC in IPF patients, suggesting a therapeutic impact on disease progression.

The specific cellular and molecular mechanisms that drive IPF are still incompletely understood. The two approved drugs, pirfenidone and nintedanib, have shown promise in reducing/managing the progression of IPF, but not in reversing the symptoms or curing the disease. A better understanding of how oxidative stress contributes to the progression of IPF may help with the identification of new targets and the development of therapeutic strategies for the protection against and treatment of IPF.

## 5. Oxidative Stress and Inflammation in Hyperoxia

Oxygen (O_2_) is a vital molecule that spreads easily in the mammalian biological microenvironment because of its lipophilicity and is essential for aerobic respiration. Oxygen also regulates the formation of ROS. It is estimated that about 1–3% of oxygen consumption is used by mitochondrial complexes I and III to generate superoxide anion (O_2_^•−^), an important signaling molecule [247] in numerous biological processes such as cell growth, proliferation, differentiation, physiological responses to oxidative stress, survival, and apoptosis [248]. Low blood O_2_ levels (hypoxemia) can be regulated by increasing the concentration of O_2_ (oxygen therapy). Excessively high levels of O_2_ (hyperoxia) can be the result of frequent iatrogenic events leading to additional O_2_ supply [249]. Oxygen therapy is included in the WHO list of essential medicines and is essential in preventing morbidity and mortality in children and adults [250]. As oxygen therapy is among the most common interventions worldwide, the consequences of hyperoxia should be carefully considered, as prolonged exposure to O_2_ in high concentrations is toxic to human health [251].

Inspired oxygen fractions are generated by an oxygen concentrator or cylinder and are commonly provided during (cardiac and thoracic) surgeries and to patients with acute respiratory syndrome to reverse/prevent hypoxemia (not dyspnea). Oxygen is delivered through tubes, facial or nasal masks (non-invasive), mechanical ventilation (invasive), or even through systems of extracorporeal membrane oxygenation to maintain arterial oxygen saturation from 94–98% (for most patients with acute illness) or 88–92% (for those at risk for hypercapnic respiratory failure) [252]. However, when a supraphysiological supply of O_2_ is maintained for a long time above an O_2_ partial pressure of ~16 kPa (120 mmHg), oxyhemoglobin saturation does not exceed 100% and results in an unnecessarily high O_2_ content in the blood [253].

Paradoxically, hyperoxia causes vast destruction of gas exchange [254], oxidative stress/damage, mitochondrial damage, inflammation, impairment of the innate immune response of macrophages to pathogens [255,256,257,258,259,260], platelet activation and sequestration in the pulmonary microcirculation [261], dysregulation of mechanisms controlling cell proliferation [262], apoptosis, necrosis, pulmonary fibrosis, and increased mortality of mechanically ventilated patients [263,264]. The airways and lungs are more vulnerable to the undesirable effects of hyperoxia because the respiratory system is the main interface for O2 delivery to the body [254]. This does not exclude the possibility that hyperoxemia induces similar systemic effects.

### 5.1. Stress and Oxidative Damage Induced by Hyperoxia

Hyperoxia alone is sufficient for the induction of oxidative stress in alveolar macrophages [265], alveolar epithelial cell types I and II [266], Clara cells [259], and pulmonary endothelial cells [267,268,269]. Oxidative stress is favored under hyperoxic conditions because the cellular redox balance is shifted towards the exaggerated formation of ROS and NOS [251].

Excess O_2_ favors the action of flavins, quinones, NADPH oxidases (especially NADPH oxidase-1) [270,271], and DUAL oxidase (lung epithelium) [272,273] by accelerating the reduction of O_2_ to O_2_^•−^, followed by the dismutation of O_2_^•−^ to H_2_O_2_ (via SOD and reactions involving Fe-S) and its reduction to HO^•^ (in the presence of Fe^2+^ and Cu^+^ ions). Higher bioavailability of O_2_ also promotes the generation of ONOO^•^ by increasing iNOS activity, accelerating the reaction between NO^•^ and O_2_ in the presence of L-arginine. Thus, ROS and NOS (and their metabolic intermediates with high reduction potential) are elevated proportionally to the concentration of excess O_2_ and should be importantly considered as inducers of pulmonary oxidative damage during hyperoxia [60,274,275,276] as they sustain oxidative stress, perpetuate lung inflammation, and induce apoptosis as well as necrosis [277,278,279,280,281]. More recent evidence also suggests that hyperoxia-induced oxidative stress may be involved in the polarization of lymphocytes during lung inflammation [282].

The disturbance of the redox balance by hyperoxia in concert with the perpetuation of oxidative stress promotes the synthesis of transcription factors sensitive to changes in the redox state, such as early growth response 1, activator protein 1, NF-κB, and Nrf2. Particularly the latter is responsible for mounting the antioxidant response [60,283,284] with antioxidant enzymes (CAT, SOD, GPx, and MPO), non-enzymatic antioxidant components (GSH, and selenium), and proteins such as HO-1 [249,285,286,287,288,289,290]. NF-κB has been recognized for its role in modulating hyperoxic inflammatory responses by stimulating IL-8 and TNF-α synthesis and high mobility group box 1 (HMGB1) [291]. Even when hyperoxia is maintained at only 50% for just 12 h, an overload of the antioxidant system can already be observed (for example, as indicated by a reduction in SOD activity). However, it is with prolonged (24–48 h) elevation of the fraction of inspired oxygen to 75–100% that this overload is marked by an intense imbalance in the activity of antioxidant enzymes (e.g., CAT, GPx, and MPO) [292,293] and glutathione depletion, which makes the lungs even more susceptible to injury [294]. Short periods of hyperoxia seem to induce an important oxidative but not an immunological response [292]. In addition, short-term hyperoxia (100% O_2_ for 2.5 h in mice and 3.5 h in humans) did not result in increased levels of inflammatory cytokines (TNF-α, IL-6, IL-8, and IL-10) in mice or healthy human volunteers [295].

The overload of antioxidant system activity and the direct attack of ROS on biomolecules lead to cellular damage in hyperoxia. For example, hyperoxia-induced oxidative stress resulted in the disruption of inner and outer mitochondrial membranes in mouse lung epithelial cells, accompanied by a reduction in aldehyde dehydrogenase activity and cytoplasm vacuolization [296]. Pulmonary oxidative damage in hyperoxia has been indirectly confirmed with the presence of lipid peroxidation products such as MDA [297], carbonylated proteins [298], and DNA base adducts (8-oxo-2′-deoxyguanosine [299]. In addition, analysis of the integrity of the epithelial cell–laminin interaction [300], the presence of ECM protein markers [301], alveolar epithelial cell number density, and lung histopathology, among other things, has indicated a reduction in the density of the vasculature [302]. The characterization of hyperoxia features using these models has directed translational controlled studies to investigate how to minimize the oxidative vulnerability of the lungs. Greater resistance to the undesirable effects promoted by hyperoxia has been reported with the exogenous administration of the NADPH oxidase inhibitors diphenyleneiodonium chloride [268], thioredoxin [303], retinoic acid [304], SOD1 [305], polyphenols (such as resveratrol) [306], isoflavonoids (e.g., formononetin) [303], aspirin [307], or surfactant [308]. Alternatively, overexpression of SOD2 and SOD3 [309,310] or mitochondrial aldehyde dehydrogenase [296], increasing the presence of catalase and HO-1 [311,312], or following an antioxidant diet have rendered beneficial effects [313]. Recently, the antioxidant effects of the calcitonin gene-related neuropeptide on type II alveolar epithelial cells were shown to be mediated by reducing MDA levels, increasing SOD activity, and activating the Notch signaling pathway [314,315].

### 5.2. Relationship between Oxidative Stress and Inflammation in Hyperoxia

Hyperoxia by itself can potentially trigger inflammation by sustaining oxidative stress and/or direct oxidative damage to alveolar macrophages (especially through apoptosis) [253], type I and type II alveolar epithelial cells [273], and pulmonary endothelial cells, in addition to inducing apoptosis in type II bronchiolar terminal and alveolar cells [277,316]. Together, these events result in a local abundance of chemokines and interleukins, which recruit leukocytes (which may or may not be super-imposed on an inflammatory scenario already established), destruction of the alveolar–capillary barrier, increased permeability, and even direct cell death [277].

Under hyperoxic conditions, alveolar epithelial cells or macrophages activate the MAPK transduction pathway, which has been associated with inflammation, death [317], and apoptosis [253]. The relationship between oxidative stress and activation of the MAPK pathway was demonstrated by exogenous exposure to H_2_O_2_ [318]. The involvement of the extracellular signal-regulated kinase (ERK) and MAPK pathways with the death of airway epithelial cells has been confirmed [272]. On the other hand, Jiang and coworkers showed that even when SMAD3 and ERK1/2 were inhibited during hyperoxia, the levels of caspase 3 increased, suggesting that the interruption of growth by apoptosis of type II alveolar epithelial cells can be compensated by other apoptotic pathways such as [255].

ERK signaling in hyperoxia is complex and can be activated from various cellular compartments, including the cytoplasm, Golgi complex, endosomes, cell membranes, nucleus, and particularly the mitochondria [284]. Under hyperoxia, activated ERK signaling can induce apoptosis and even sustain macrophage viability in hyperoxia-inflamed lungs [319]. In parallel, NADPH-induced ROS generation in hyperoxia activates small GTPases [320,321], which in turn activate the MAPK pathway and c-Jun NH2-terminal kinase (JNK) [322], resulting in increased expression of TGF-β1 and production of TNF-α, IL-1β, IL-6 and IL-8 [286,323,324]; the latter has a strong correlation with neutrophilic influx [325]. Paradoxically, the presence of TNF-α, IL-1β, and IL-6 has been linked to longer survival of mice exposed to hyperoxia and inflammation, probably due to their broad roles as autocrine, paracrine, and exocrine inflammatory mediators [325]. Early evidence suggested that the presence of TNF-α and IL-1β offered protection against hyperoxic lung injury by a feedback mechanism involving an upregulation of the antioxidant response with increased SOD2 activity [326]. However, it is now known that TNF-α and IL-1β induce strong expression of IL-6 [327], a pleiotropic cytokine produced at inflammatory sites that induces lymphocyte (B and T) recruitment or even triggers anti-inflammatory responses. IL-1β is also known to recruit cells to the site of inflammation and stimulate the production of pro-inflammatory mediators [325]. Evidently, the involvement of cytokines in hyperoxia is complex and seems to be related to the experimental model and the time of exposure to O_2_. For example, IL-1β was not elevated after 12 h of hyperoxia [328] but increased after 24 h [329], 48 h [330], 3 days [331], 4 days [332], and 7 days [333].

The role of IL-6 appears to be controversial in hyperoxia. Ward and co-workers showed that IL-6 protects the lungs from hyperoxic injury as evidenced by reductions in cell death and DNA fragmentation due to the induction of B-cell lymphoma 2 protein and the presence of TIMP-1 [327]. In neonatal mice, the pulmonary inflammatory response to hyperoxia was characterized by IL-6, TNF-α, IL-12, IL-10, and MCP1, but the inflammatory response and pulmonary cell death were significantly enhanced in the absence of IL-6 [334]. On the other hand, there is clear evidence that IL-6 is involved in acute cases of hyperoxia-induced lung inflammation and oxidative stress [293], in addition to the polarization of T cells to the Th17 phenotype, especially when hyperoxia is superimposed on a pre-existing inflammatory scenario [282]. The pattern of the acute inflammatory response in an experimental murine model of asthma was modified by hyperoxia to a Th17 phenotype, characterized by an intense neutrophilic infiltrate and the presence of IL-17 [282]. This type of immune response has received special attention in experimental models of lung inflammation because it is recognized as an acute, complex, and difficult-to-control response, and can be further promoted by the local release of IL-17 and IL-22 from Th17 [335,336]. In addition to the presence of IL-6, hyperoxia by itself induces the release of TGF-β1 [337], and the expression of T-box expressed in T cells (Tbet) has been confirmed [282]. This repertoire of molecules in hyperoxia is specifically important because activation of their associated receptors is necessary for the differentiation of naive Th cells into Th17 cells [338,339,340].

Activation of NF-κB leads to increased expression of intercellular adhesion molecule 1 (ICAM-1) and platelet endothelial cell adhesion molecule 1 (PECAM-1) in lung endothelial cells and facilitates neutrophilic adhesion as well as transmigration in hyperoxia [341,342]. Neutrophils are important sources of ROS and MMPs, and neutrophilic influx during hyperoxia has been linked to DNA damage in rat lungs [343]. It was observed in mice that even with attenuation of neutrophil activity, lung damage was still maintained [274]. As indicated, hyperoxic lung inflammation is complex and involves various other cells, including macrophages, endothelial cells, alveolar epithelial cells, and platelets [261]. Alveolar macrophages are normally resistant to injury but are subject to apoptosis under hyperoxia, during which activation of the ERK/MAPK pathway seems to lead to macrophage death [253]. There is evidence to suggest that even under intense hyperoxia-induced oxidative stress with a marked increase in SOD, CAT, and glucose-6-phosphate dehydrogenase activity, macrophages manage to survive and proliferate for up to 48 h; however, after 60 h of high oxygen exposure proliferation is suppressed [344], and prolonged oxidative stress results in cell death [345]. A sustained activation of the ERK pathway maintains macrophage survival under hyperoxia by reducing the action of the pro-apoptotic protein Bcl-2 by downregulating specific phosphatases [319].

Macrophages are the most important sources of ROS in hyperoxia [265] and, when active in the hyperoxygenated microenvironment, sustain oxidative stress/damage, and inflammation. Alveolar macrophages respond to the initial insult of hyperoxia by synthesizing and secreting (and being self-stimulated by) IL-1β, IL-6, TNF-α, interferon-gamma [331,346], HMGB1 [291] and eicosanoids derived from cyclooxygenase and 5-lipoxygenase (e.g., leukotriene B4) [347]. Both HMGB1 [348] and leukotriene B4 [349] induce neutrophil influx into the lungs. Hyperoxia-stimulated alveolar macrophages recruit neutrophils to the lungs via the activation of chemokine receptors [350] and produce and release factors that increase neutrophilic adherence [301]. In newborn infants, hyperoxia induces macrophage polarization to the M1 phenotype, favoring the presence of IL-6/STAT3 in the lungs [351]. Experimental models show that high levels of IL-6 (but also TNF-α and IL-1β) in the lungs are directly related to longer exposure to hyperoxia [293,331,333,352].

Endothelial injury and epithelial death are key points in the pathogenesis of hyperoxia-induced lung inflammation by directly impairing the integrity of the alveolar—capillary barrier. Bhandari and co-workers reported that extrinsic and intrinsic pathways of cell death, including necrosis, can be observed in hyperoxia. These authors showed that the elevated expression of angiopoietin 2 increased the expression of caspases 3, 8, and 9 [353]. Additionally, dysfunction of the alveolar–capillary barrier and alveolar macrophage death are associated with K+ efflux through the P2X7 receptor that keeps inflammasomes and cytokine secretion activated [352]. Hyperoxia-induced circulating extracellular vesicles containing high amounts of gasdermin Dp30, an inflammasome-mediated cell death executor protein that may contribute to the pathological features of bronchopulmonary dysplasia, have been demonstrated in neonatal rats [354]. Using an in vitro murine cell culture system, a short period of hyperoxia was shown to promote a disturbance in N-glycolysis, especially in lung endothelial cells, suggesting a potential impairment of receptor signaling on the endothelial inner surface and interaction with immune cells [355].

Approximately four decades ago, Barry and Crapo pointed to the massive sequestration of platelets in the pulmonary microcirculation of rats as a potentially important event in hyperoxia [261]. Barazzone and co-workers later showed that this phenomenon is dependent on TNF-α and CD11a [356]. Unfortunately, in-depth studies investigating the contribution of platelets in lung injury induced by hyperoxia are lacking. It is known that macrophages under hyperoxia activate the extrinsic pathway of coagulation by binding cell surface lipoproteins to fibrinogen in contact with the interstitium when epithelial damage is induced [357]. Future studies are warranted to help clarify whether hyperoxia is related to thrombotic clinical events, especially considering O_2_ is normally superimposed on a pre-existing inflammatory condition.

The airway epithelium regulates hyperoxic pulmonary inflammation by stimulating IL-11 gene expression by inducing IL-1α, TGF-β1, and TGF-β2 [323,358], through the synthesis of surfactant [308], and perhaps by the expression of IL-10. To date, the role of IL-10 in the response of the lungs to hyperoxia remains inconclusive [325]. TGF-β has received considerable interest as it increases collagen I production [359], drives fibroblast differentiation into myofibroblasts, blocks alveolarization, and promotes differentiation of naive T cells into Th17 cells [282,360]. In response to hyperoxia-induced lung injury, fibroblasts migrate to the lungs, proliferate, and differentiate into myofibroblasts that produce collagen type I (rather than collagen III), thereby modifying the ECM [359]. The early deposition of type I collagen, in parallel with inflammation, has contributed to the “new” concept of bronchopulmonary dysplasia, characterized by delayed alveolar development, sustained inflammation, mild fibrosis, and damage to the integrity of the alveolar–capillary barrier [361]. Hyperoxic collagen I synthesis in lung fibroblasts has been linked to oxidative stress and the consequent activation of the Rho-associated protein kinase pathway [320].

The identification of inflammatory and cell death pathways activated in hyperoxia has led to different studies on how to attenuate the impact of these pathways on lung damage. Administration of FGF-18 inhibited the NF-κB signaling pathway and reduced oxidative stress and inflammation levels in type II epithelial cells exposed to hyperoxia [362]. Overexpression of clusterin, a glycoprotein expressed in airway epithelial cells, protected against hyperoxia-induced mitochondrial membrane disruption and prevented BCL2-associated X apoptosis regulator protein translocation, cytochrome C release, and caspase activation [363]. Inhibition of the ERK pathway by semaphorin 3A suppressed hyperoxia-induced apoptosis and inflammation [364]. Administration of chrysin (a flavonoid) attenuated lung damage via increased antioxidant activities (SOD and GSH), suppression of pro-inflammatory mediators (TNF-α and IL-1β), improving alveolarization, and reducing apoptosis (caspase 3) in an experimental model of hyperoxia-induced lung injury [365].

Several other approaches have been successful against oxidative stress and lung inflammation induced by hyperoxia. Bezerra and co-workers demonstrated that treatment with exogenous surfactant reduced oxidative stress and inflammation markers (HMGB1, TNF-α, and IL-17) in lung tissue homogenates obtained from adult mice [308]. Others have indicated the beneficial effects of etomidate on hyperoxia-induced stress and inflammation in mice with upregulation of Nrf2 and HO-1 in lung tissue [366]. In neonatal rats exposed to hyperoxia, administration of 18β-glycyrrhetinic acid (the major metabolite of glycyrrhizin) inhibited the activation of NF-κB and the NLRP3 inflammasome and decreased ROS levels [367]. Treatment with fucoidan (a class of L-fucose-enriched sulfated polysaccharides complex) protected newborn rats from hyperoxia-induced lung injury by suppressing cell apoptosis, mitigating oxidative stress, and inhibiting the differentiation of lung fibroblasts into myofibroblasts, thereby reducing lung fibrosis and collagen I deposition [359].

**Figure 5:**
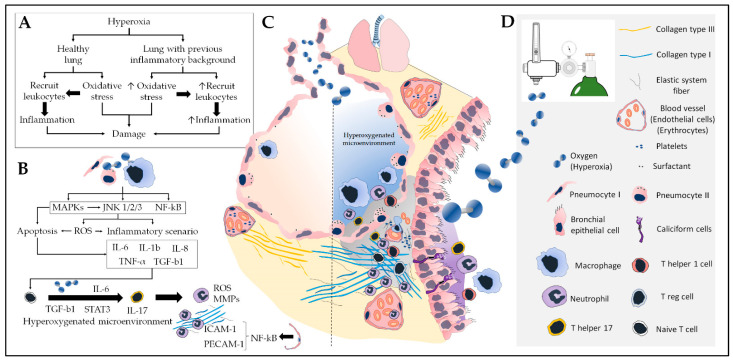
The early phase of hyperoxia-induced oxidative stress and inflammation. (**A**) Hyperoxia in healthy lungs results in oxidative stress-induced lung damage and inflammation. When hyperoxia is superimposed on lungs with pre-existing inflammation, oxidative stress-induced lung damage and inflammation are amplified. (**B**) Hyperoxia activates MAPK, JNK, and NF-κB pathways in macrophages and lung epithelial cells, leading to apoptosis, oxygen-free radical formation, and an inflammatory microenvironment with a cytokine repertoire (IL-6, IL-1β, IL-8, TNF-α, TGF-β1, and STAT3) that favors polarization of naïve T cells to Th1 and Th17, and promotes neutrophil attraction. Endothelial cells express ICAM-1 and PECAM-1 for neutrophil adhesion. (**C**) Hyperoxia-induced oxidative stress as well as damage to macrophages and lung epithelial cells lead to increased vascular permeability, elevated mucus production, and robust recruitment of lymphocytes, neutrophils, and macrophages to the inflammatory site. Endothelial damage scatters erythrocytes and promotes platelet aggregation on extracellular matrix proteins. (**D**) An example of supraphysiological oxygen delivery by Thorpe and Bourdon tubes.

Here, we have outlined that hyperoxia results in endothelial damage, alveolar epithelial damage, increased blood vessel permeability, edema, hypercellularity, and activation of chemotactic factors/interleukins, cumulatively resulting in lung morphological changes (see Figure 5). Oxidative damage induced by hyperoxia can occur by concomitant oxidation of cellular and extracellular biomolecules, caused by the attack of ROS and RNS formed directly from the excess oxygen available. In addition, proteolytic enzymes from inflammatory cells, particularly neutrophils and macrophages, contribute to lung remodeling. Because the lungs suffer from the consequences of various insults, such as mechanical stress, infections, hypoperfusion, systemic inflammation, and drug toxicity, the relative role of hyperoxia in the induction of lung damage in humans is difficult to assess, especially considering most patients who are subjected to hyperoxic conditions present with background (often pulmonary) diseases. Therefore, we strongly believe that continued studies on the “oxidative burst” induced by hyperoxia, especially when superimposed on a pre-existing inflammatory condition, are required. A better understanding of the molecular and cellular events involved in hyperoxia-induced lung injury will help to prevent/limit the iatrogenic consequences of O_2_ treatment.

## 6. Oxidative Stress and Inflammation in Sepsis

Oxidative stress and inflammation play a major role in sepsis development. Following the Third International Consensus Definitions for Sepsis and Septic Shock meeting in 2016, sepsis is defined as “a life-threatening organ dysfunction caused by a dysregulated host response to infection” [368]. Based on data from the Global Burden of Diseases, the incidence of sepsis in 2017 was estimated at 48.9 million worldwide, and 11.0 million sepsis-related deaths were recorded, representing a total of 19.7% of deaths registered globally [369]. In the USA, sepsis was the hospital’s most costly condition, accounting for more than USD 24 billion and representing 13% of American hospital costs [370].

Sepsis is a complex and multifactorial syndrome in which the host’s immune response may vary considerably. For instance, the causative pathogen and the site of infection are critical for the development of the host response. Infection can be caused by various sources, such as bacteria, viruses, fungi, and parasitic pathogens. Among bacteria, Gram-negative species are responsible for more sepsis cases than Gram-positive species [371]. There are many different sites of primary infection, with the lungs being the most common with regards to sepsis-related death [372].

### 6.1. Sepsis and Acute Lung Injury/Acute Respiratory Distress Syndrome

Lung infections such as pneumonia can be followed by sepsis. This can develop into direct sepsis-induced acute lung injury (ALI) or its most severe variant, acute respiratory distress syndrome (ARDS). Sepsis can also indirectly generate ALI/ARDS when the primary infection occurs outside the lungs (e.g., in the gastrointestinal tract) [373]. ALI and ARDS are characterized by vascular leakage, alveolar exudates, and hypoxemia, followed by a fibroproliferative stage that can either be turned off and resolved or evolve into fibrosis and ultimately death [374].

Following infection, there is a disruption of lung endothelial and epithelial barriers, leading to increased permeability to liquid and proteins, causing edema in the alveoli. In addition, there is excessive recruitment and accumulation of neutrophils, monocytes, inflammatory cytokines, and red blood cells in the alveolar space. Although inflammation is important for pathogen clearance and disease resolution, its excessive activation may have detrimental effects such as tissue damage. The exaggerated inflammatory response can damage alveolar epithelial cells by inducing increased p53 (tumor suppressor gene) expression and subsequent apoptosis [375]. Sepsis is also characterized by a cytokine storm, consisting of an uncontrolled production of pro-inflammatory cytokines such as TNF-α, IL-6, and IL-1β [376,377]. These cytokines can, among other things, induce the activation of endothelial cells, resulting in an upregulation of adhesion molecules (e.g., P- and E-selectin), which will lead to an increased deposition of neutrophils into the alveolar spaces. Activated platelets also accumulate in lung tissue by interacting with neutrophils (neutrophil platelet aggregates) [378]. These aggregates contribute to lung injury as it has been shown they have a greater phagocytic capacity, adhesive function, and ability to produce toxic oxygen metabolites [379].

Leukocytes play a key role in the pathogenesis of sepsis. In addition to the massive influx of neutrophils, other important cells include resident alveolar and recruited (from the blood) macrophages [380,381,382]. Neutrophils are the first cells to be recruited to the site of infection, and their accumulation into the lung spaces is a hallmark of septic inflammation. The number of neutrophils in the BAL was greater in patients with sepsis-induced ARDS who died compared to those who survived. However, neutrophil numbers did not serve as a death predictor for patients with ARDS due to trauma and other causes [383].

**Figure 6:**
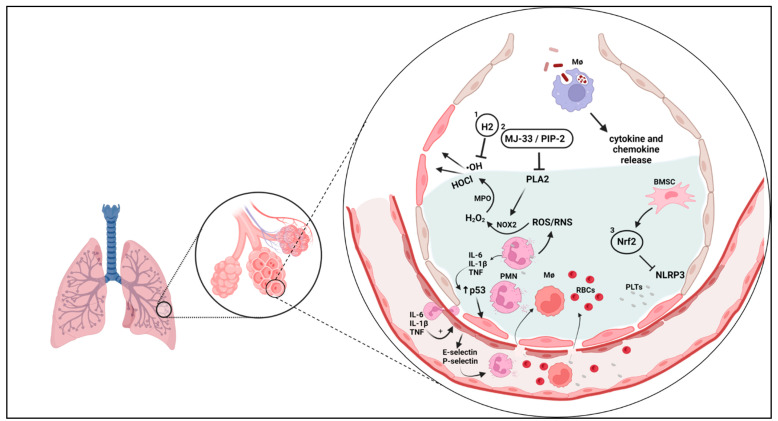
Schematic representation of a damaged alveolus—lung tissue can be damaged either directly or indirectly through exacerbated inflammation in other organs. In direct injury, resident alveolar macrophages recognize invading pathogens through pattern recognition receptors (not shown) present on their surfaces, producing cytokines and chemokines. Neutrophils are recruited to alveolar spaces, where they produce more cytokines and ROS/RNS, leading to endothelial and epithelial damage. Increased epithelial cell apoptosis through augmented expression of p53 also occurs and will disrupt the tight barriers, resulting in an increase in edematous fluid and RBCs, as well as neutrophil infiltration into alveolar tissue. Neutrophils migrate towards increased expression of selectins (on endothelial cells) and can form aggregates with platelets. Many neutrophils will produce excessive reactive oxygen metabolites through the expression of NOX2, and MPO will ultimately convert H_2_O_2_ into its final products. Antioxidant strategies are thought to decrease excessive ROS production through (1) the neutralization of oxygen metabolites, (2) inhibiting PLA2, leading to NOX2 inhibition, or (3) inducing Nrf2 activation (using BMSC). Abbreviations: RBCs, red blood cells; NOX2, NADPH oxidase 2; MPO, myeloperoxidase; H_2_O_2,_ hydrogen peroxide; PLA2, phospholipase A2; Nrf2, nuclear factor erythroid 2-related factor 2; BMSC, bone marrow-derived mesenchymal stem cells.

ROS and RNS production are important mechanisms of the innate immune system to combat invading pathogens due to their antimicrobial function. However, the dysregulated production of ROS and RNS can contribute to ARDS progression through lung tissue damage. Neutrophils contain the NADPH oxidase complex and, as such, are an important source of ROS [384,385]. This complex can reduce oxygen into a superoxide anion that will be released outside the cell and then dismutated into H_2_O_2,_ a more stable product. H_2_O_2_ can further be catalyzed into hydroxyl radicals and HOCl by MPO, an enzyme present in neutrophil granules [386]. Excessive ROS production can damage cells in many ways. For example, lung epithelial cells were damaged by ROS through a reaction with DNA, altering cellular structure and function [387]. Furthermore, ROS can cause oxidative stress through the peroxidation of arachidonic acid present in cell membranes [388] and contribute to inflammation by inducing the activation of transcription factors that will lead to inflammatory mediator production [389]. A classical study by Johnson et al. using a rat model has greatly contributed to the understanding of ROS toxicity in the lung by showing that intrapulmonary instillation of enzymes that generate oxygen metabolites results in ALI [390]. Under physiological conditions, the antioxidant defense system plays an important role in quenching ROS/RNS, thereby preventing tissue damage [391]. However, in ALI/ARDS disease, there is a redox imbalance favoring ROS production rather than scavenging. Accordingly, increased plasma lipid peroxidation products and decreased antioxidant plasma levels have been reported in ALI/ARDS patients [392]. Thus, ROS plays an important role in the onset of inflammation and the progression of ALI in sepsis, which makes them a potential therapeutic target. The general events linked to lung infection followed by exacerbated ROS production and inflammation culminating in ALI/ARDS development and lung sepsis are summarized in Figure 6.

### 6.2. Antioxidant Treatment in Sepsis

Among the many available models to study sepsis, three stand out: (I) endotoxemia (systemic inoculation of LPS), (II) bacterial inoculation (systemic administration of *Escherichia coli*, for example), and (III) barrier-disrupting manipulations (such as cecum perforation, CLP—cecal ligation and puncture). The latter is currently considered the gold standard, as it represents a condition similar to the clinical course of sepsis, presenting pathophysiological changes over time in different organs and tissues [393]. Using these experimental models, the important role of antioxidants in dampening damage caused by excessive ROS production has been demonstrated. For example, molecular hydrogen (dihydrogen, H_2_) has been shown to diminish the harm caused by OH^−•^ through neutralization [394]. Indeed, in a model of LPS-induced ALI, rats treated with hydrogen-rich saline exhibited attenuated lung inflammation, reduced macrophage apoptosis, and increased macrophages with an M2 phenotype [395].

As indicated earlier, Nrf2 is an important transcription factor involved in the oxidative stress response. Under moderate oxidative stress, Nrf2 is activated and translocated to the nucleus, where it can act as a transcriptional activator of antioxidant genes. Studies using a mouse model of LPS-induced ALI showed protective effects of bone marrow-derived mesenchymal stem cells (BMSC), as evidenced by a decrease in inflammatory and oxidant effects in the lung because of inhibited inflammasome NLRP3 signaling. This beneficial effect was partially disrupted by Nrf2 inhibitors, suggesting that Nrf2 inhibition can increase levels of ROS, which in turn can provide essential signals for NLRP3 activation [396]. Phospholipase A2 (PLA2) is an important enzyme that participates in the activation of NOX2 and the production of ROS. In a model of LPS-induced ALI, the non-specific cPLA2 inhibitor, MJ-33, protected mice against lung injury [397]. The same group used PIP-2, a peptide derived from surfactant protein A (a natural lung protein), which inhibits PLA2 activity, and showed that the inhibition of PLA2/NOX2 signaling by PIP-2 was effective in preventing ALI in a model with bacterial infection [398]. Some clinical trials using antioxidants for the treatment of sepsis have been carried out. It has been shown that plasma levels of the potent antioxidant vitamin C decrease under some stress conditions such as infections [399]. Administration of vitamin C shortened the length of stay in the ICU and the duration of mechanical ventilation in sepsis patients [400,401]. However, the use of vitamin C is still debatable considering some studies did not find a positive result [402]. Because oxidative stress plays an important role in ALI/ARDS development in sepsis, exploring treatment strategies involving different antioxidants could be of therapeutic relevance.

## 7. Oxidative Stress and Inflammation in Ventilator-Induced Lung Injury (VILI)

Mechanical ventilation (MV) is an invasive life support tool used in ICUs for patients at risk of dying Although MV saves millions of lives per year, lung expansion and contraction caused by MV promote inflammatory cell infiltration and oxidative stress that may result in ventilator-induced lung injury (VILI) [403]. The physical mechanisms associated with VILI are alveolar overdistension due to high lung volumes and collapsing, i.e., reopening the functional units of low-volume ventilated lungs [404,405,406]. When there is an association between high lung volumes and high airway pressure with increased vascular permeability, MV also promotes damage to the lung parenchyma, both in experimental and clinical studies [407,408,409,410]. Thus, mechanical stretching triggers a pulmonary inflammatory response through the recruitment of cells and the release of inflammatory mediators, as well as the production of local and systemic ROS, which is collectively referred to as biotrauma [403,411,412]. Alveolar epithelial cells (AEC), macrophages, and neutrophils are the main cells responsible for the pulmonary inflammatory response induced by VILI [413,414,415].

Mechanotransduction caused by MV might damage cellular membranes via Ca^2+^, specifically in AECs [415]. MV with high tidal volume can trigger the release of proinflammatory mediators such as IL-1β, IL-6, IL-8, TNF-α, CXCL1, CXCL10, and MIP-2 in the BAL in mice [416,417,418]. Among these, IL-6 and IL-1β were also found to stimulate NF-κB activation [416]. Recently, an experimental study in rats demonstrated that high-pressure ventilation increased IL-33 levels in lung tissue, suggesting IL-33 as a new biomarker of VILI [419].

HMGB1 is a non-histone nuclear protein and damage-associated molecular pattern molecule expressed by AEC and alveolar macrophages; it attracts neutrophils and can be associated with VILI [308,420,421]. Another experimental study revealed that HMGB1 accumulated in the BAL obtained from rabbits subjected to MV [422]. During VILI, extracellular HMGB1 binds to TLRs and receptor for advanced glycation end (RAGE) products, which activates intracellular p38 and other downstream signaling pathways in ALI [423].

Regarding alveolar macrophages, MV with a high tidal volume has been shown to promote autophagy and HMGB1 secretion in cells obtained from mouse lung, thereby increasing the pulmonary inflammatory response [423,424]. Other experimental models demonstrated neutrophils recruited from the blood to the airways (through the endothelium via gap junctions) as important cells contributing to VILI [404,412,414,421,425,426]. The activation of neutrophils is initiated by proinflammatory cytokines, such as TNF-α and IL-1β, after which neutrophils generate and release NETs that have been shown to act pathogenic in mouse VILI [427]. TLR4 is a regulator of neutrophil activation and survival; accordingly, the TLR4-MyD88 signaling pathway plays a key role in the pathogenesis of VILI with high tidal volume in mice [427]. Once recruited into the lung parenchyma, neutrophils release granules that may contain MPO, NADPH oxidase, and MMP9/neutrophil elastase. These enzymes are vital in the production of ROS as well as the activation of ECM degradation in the lung parenchyma in response to MV [404,411,428].

Cyclic stretch caused by MV promotes the activation of the NADPH oxidase system, which consists of transmembrane and cytosolic proteins that belong to the NOX family. The subunits gp91phox (NOX2) and p47phox are essential for the production of ROS such as hydrogen peroxide H_2_O_2_, O_2_**^•^**^−^ and ^•^OH induced by cyclic stretching in VILI [391]. RNS (nitric oxide NO^•^ and its metabolites) also have an important role in experimental models that use high tidal volumes to induce VILI [404]. NO^•^ can be generated by three isoforms of NOS (neuronal, endothelial, and inducible). The iNOS isoform has been directly associated with VILI in a rat model [429]. The breakdown of the redox status caused by the excessive production of RNS and ROS under MV causes dysregulation in the production of the endogenous antioxidant defense system [391]. The response to oxidative insults promoted by MV-induced cyclic stress is mainly mediated by the antioxidant response element (ARE). Thus, Nfr2 induces transcription and translation of pulmonary antioxidant enzymes/proteins in response to a pro-oxidant stimulus. Under basal conditions, Nrf2 is bound to keap1 and maintained in the cell cytoplasm. However, under cyclic stretch induced by MV, Nfr2 disconnects from Keap1 and translocates to the nucleus inducing the transcription of several genes associated with detoxification and cytoprotection [391].

**Figure 7:**
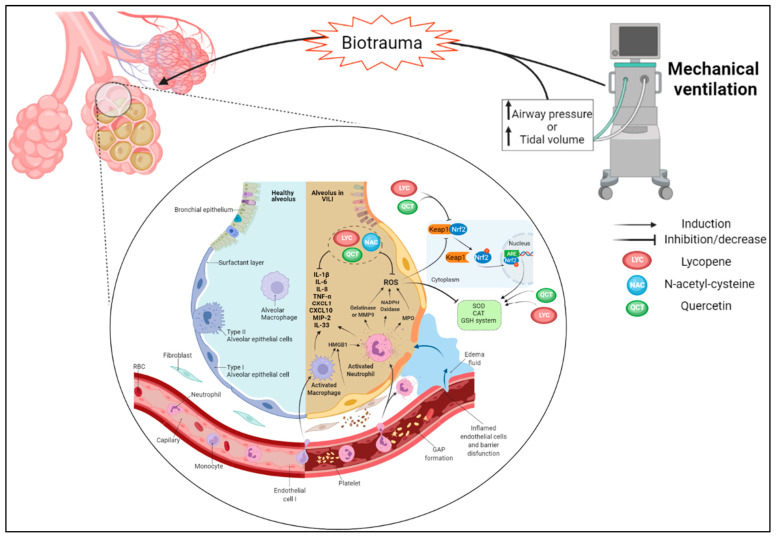
Overview of the mechanisms associated with ventilator-induced lung injury (VILI) and proposed bioactive compounds that may prevent or treat VILI caused by mechanical ventilation (MV). Abbreviations: antioxidant response element (ARE); catalase (CAT); C-X-C motif ligand 1 (CXCL1); CXCL10; glutathione system (GSH system)); high mobility group box 1 (HMGB1); interleukin (IL) 1β; IL-6; IL-8; IL-33; Kelch-like ECH-associated protein 1 (keap1); macrophage inflammatory protein 2 (MIP-2); matrix metalloproteinase 9 (MMP9); myeloperoxidase (MPO); nuclear factor erythroid 2-related factor 2 (Nfr2); reactive oxygen species (ROS); red blood cell (RBC); superoxide dismutase (SOD); tumor-necrosis factor (TNF)-α.

Our research group has explored various experimental models of VILI (high tidal volume, positive end-expiratory pressure, volume-controlled, or pressure-controlled) using healthy animals. We observed an increase in the antioxidant enzymes SOD and CAT, a depletion of the glutathione system associated with an increase in oxidative damage to lung tissue, an increase in lipid peroxidation and levels of carbonylated protein, and an increase in MPO activity. All these alterations were paralleled by the recruitment of inflammatory cells and the production of important cytokines and chemokines induced by MV [404,411,412,421,425]. In experimental models of VILI, the antioxidant NAC was capable of reducing acute lung injury caused by MV [391]. Recently, some other bioactive compounds have been suggested to protect against or prevent VILI induced by MV. In adult rats subjected to mechanical ventilation (high tidal volume of 25 mL/kg for 4 h), treatment with various concentrations of the polyphenol curcumin ameliorated the MV-induced redox imbalance and inflammation, likely involving inhibition of NF-κB activation [430]. Our group has worked with the bioactive compound lycopene—a carotenoid responsible for the red color of various foods such as tomatoes, watermelon, papaya, pink grapefruit, and guava—with nutraceutical (i.e., both pharmacological and nutritional) properties. Lycopene acts by reducing the redox imbalance, increasing antioxidant enzyme levels, as well as improving immune response through cell cycle regulation and modulation of gene expression [431]. Therefore, we speculate that nutraceutical compounds such as lycopene and other antioxidants (e.g., quercetin [432], hesperidin [433]) may represent new therapeutic alternatives to prevent or treat VILI (Figure 7). These speculations need to be (further) validated both in preclinical experimental models and clinical studies.

## 8. Conclusions

This review addressed the respiratory conditions that most affect the airways and lungs: COPD, IPF, sepsis, VILI, asthma, and hyperoxia. The inflammatory responses in each of these conditions are specific and rely on the participation of multiple cytokines/chemokines and signaling mechanisms. Generally, transcription factors such as NF-κB and AP1, as well as certain kinases (e.g., MAPK), are involved in inflammation. All inflammatory responses converge on the accumulation and activation of inflammatory cells, such as macrophages, eosinophils, neutrophils, and lymphocytes, with subsequent damage and/or (phenotypic) modulation of airway resident cells (e.g., epithelial and mesenchymal cells). Despite the peculiarities of each pathological condition, the inflammatory and tissue responses culminate in physiological damage that, especially when persistent, could (chronically) impair lung function. Inflammation often generates an oxidative stress response. This holds true particularly in the lungs, where continuous exposure to air and (high concentrations of) oxygen provide epithelial, mesenchymal, endothelial, and inflammatory cells with a rich and abundant substrate for redox signaling. In this context, all respiratory conditions discussed here have a redox-signaling component.

Current drugs targeting respiratory diseases often treat symptoms, providing temporary relief without directly acting on the cause. As outlined in this review, antioxidants have shown great potential in the treatment of various respiratory conditions associated with oxidative stress, as evidenced by their efficacy in different experimental lung disease models and a few clinical studies. To date, the vast majority of promising results obtained using animal models have not been reproduced in clinical trials. However, given the pharmacological safety, limited side effects, and the continuous emergence of novel potent compounds, antioxidants should be further explored as therapeutic agents for the treatment of respiratory diseases.

## Figures and Tables

**Table 1:** Application of synthetic antioxidants (AO) for the treatment of emphysema in animal models.

Synthetic AO against Emphysema	Outcome Observed	References
N-acetylcysteine	Preserves GSH/GSSG, anti-inflammatory, reduces exacerbations	[186,187,188,189]
Aminoguanidine	Reduces peroxynitrite and tissue damage	[190]
MnTE-2-PyP	SOD3 mimetic reduces extracellular matrix damage	[191]
Diallyl disulfide	Prevents tissue and oxidative damage, inhibits cytokine production, and potentiates antioxidant enzymes.	[196,197]
Statins	Reduces ROS, inhibiting tissue as well as oxidative damage, and activates Nrf2 and AO enzymes.	[199,200,202]

**Table 2:** Application of natural antioxidants (AO) for treating (features) of emphysema in cell systems (*) and animal models.

Natural AO against Emphysema	Outcome Observed	Reference
Mate tea (chlorogenic acid main polyphenol)	Reduces MPO activity; prevents oxidative and tissue damage; increases CAT activity and GSH/GSSG	[203]
Eucalyptol	Reduces oxidative and tissue damage; increases SOD activity	[204]
Propolis	Induces pro-resolutive macrophages; reduces tissue damage	[205]
Quercetin	Reduces oxidative damage; suppresses iNOS, cytokines, MMPs; increases AO enzyme activity	[208,209]
Resveratrol	Ameliorates lung function; activates Nrf2, SOD, Sirt1; reduces oxidative damage and cytokines	[88,211]
Curcumin	Induces autophagy; reduces ER stress by Sirt1 activation; reduces cytokines; ameliorates lung function	[212,213]
Sulforaphane	Upregulates Nrf2; reduces ROS, oxidative damage, and inflammatory pathways involving TLR2, TLR4 and Myd88 *	[214,215]
Vitamin C	Reduces oxidative and tissue damage; increases collagen synthesis and vascular endothelial growth factor levels	[217]
Vitamin E	Reduces oxidative and tissue damage as well as inflammation; upregulates Nrf2 and AO enzyme activity	[218]
